# Urban greening resources and visibility–soil-moisture coordination in Dubai: Spatiotemporal patterns, spatially varying drivers, and planning implications

**DOI:** 10.1371/journal.pone.0360918

**Published:** 2026-10-23

**Authors:** Jianyi Li

**Affiliations:** China Urban Construction Design & Research Institute C.O., LTD, Beijing, China; Wallaga University, ETHIOPIA

## Abstract

Urban greening in desert cities is closely related to atmospheric visibility and near-surface water conditions, yet its environmental performance depends on vegetation condition, landscape configuration, atmospheric context, and irrigation demand. Taking Dubai as the study area and 2010, 2015, and 2020 as reference years, this study integrates Landsat imagery, GLC_FCS30D land-use data, atmospheric visibility, precipitation, and surface soil moisture to examine the spatial and temporal coordination between visibility-related atmospheric conditions and water availability. It further analyzes the changes in the Normalized Difference Vegetation Index (NDVI), Fractional Vegetation Cover (FVC), Leaf Area Index (LAI), and the landscape patterns of different land-use types. An optimal-parameter geographical detector, ordinary least squares regression, and geographically weighted regression are used to identify the stage-dependent and spatially varying associations between urban greening resources and environmental coordination.The principal finding is that the relationship between urban greening resources and the visibility–soil-moisture coordination index was both stage-dependent and spatially nonstationary. FVC was the dominant individual factor in 2010 and 2020, whereas ED dominated in 2015, while the direction and magnitude of local effects varied among coastal built-up areas, urban expansion fringes, and inland desert areas. The results further show that: (1) Mean visibility generally declined during the study period, whereas surface soil moisture decreased from 2010 to 2015 and subsequently recovered from 2015 to 2020. Areas with low coordination gradually shifted from the southeastern and southern parts of the study area towards the central urban area, indicating a spatial mismatch between atmospheric visibility and surface water conditions. (2) From 2010 to 2020, the proportion of bare land decreased by 2.88 percentage points, whereas impervious surfaces and managed vegetation increased by 1.72 and 1.56 percentage points, respectively. The edge density and patch density of managed vegetation increased continuously, indicating that the expansion of green spaces was accompanied by increasing landscape fragmentation. During the same period, NDVI increased by a dynamic degree of 1.15%, whereas FVC decreased by 0.93% and LAI changed only slightly, with a dynamic degree of −0.08%. These results indicate that local vegetation greenness improved without a corresponding increase in continuous vegetation coverage or canopy structure. (3) The explanatory power of the driving factors was strongly stage-dependent. FVC was the dominant factor in 2010 and 2020, whereas edge density was dominant in 2015. The interaction effects between vegetation indicators and landscape-pattern metrics were generally stronger than the effects of individual factors, indicating that vegetation condition and landscape structure jointly differentiated the coordination state. (4) The coefficients of FVC, edge density, and patch density varied substantially across locations and years, demonstrating spatially varying associations between urban greening resources and environmental coordination. These findings suggest that urban greening should be planned according to local vegetation conditions, landscape structure, atmospheric visibility, ventilation requirements, and water availability. Coastal built-up areas, urban expansion fringes, inland areas, and central urban districts should therefore adopt differentiated vegetation configurations and irrigation strategies. This study provides a basis for coordinating urban greening, atmospheric-environmental conditions, landscape structure, and water-resource allocation in arid cities.

## 1. Introduction

Against the backdrop of intensifying global climate change and land degradation, sand and dust storms (SDS) have become one of the most significant environmental risks in arid and semi-arid regions [1,2]. SDS are typically triggered by strong winds, which entrain fine surface particles into the atmosphere through wind erosion. These particles can subsequently be transported across regions or even continents by large-scale atmospheric circulation, thereby exerting substantial impacts on the global environmental system. According to an assessment by the United Nations Environment Programme, SDS are not only a typical meteorological hazard but also an important manifestation of the interaction between global land degradation and climate change. At the global scale, SDS are primarily concentrated within the so-called “global dust belt,” which extends broadly from North Africa and West Asia to Central and East Asia and contains the world’s major dust-source regions. Nevertheless, long-range atmospheric transport can carry dust particles thousands of kilometres, affecting cities and ecosystems far from their source areas [3]. Previous studies have shown that dust aerosols not only alter the atmospheric radiation balance but also affect cloud formation, precipitation processes, and biogeochemical cycles, making them an important component of the global climate system.

A substantial body of research has demonstrated that dust activity has undergone marked changes in several arid regions worldwide in recent decades [4]. Climate warming, declining precipitation, and vegetation degradation can increase the extent of exposed land and reduce soil moisture, thereby intensifying wind erosion and increasing dust-emission intensity. For example, Goudie and Middleton noted in their seminal study that changes in SDS frequency may represent an important manifestation of global environmental change while also further destabilising the climate system, thereby creating a feedback mechanism [5]. Meanwhile, the impacts of SDS on human societies have received increasing attention. Epidemiological evidence indicates that airborne particulate-matter concentrations rise substantially during SDS events, potentially triggering or aggravating respiratory and cardiovascular diseases. A systematic review of more than 200 studies worldwide found that over 84% reported significant associations between SDS exposure and adverse health outcomes, including increased morbidity and mortality from respiratory diseases [6]. SDS also exert wide-ranging effects on socioeconomic systems. Dust deposition can reduce agricultural productivity, damage infrastructure, and disrupt transportation and urban operations by impairing visibility. Relevant research has shown that increases in SDS frequency may reduce agricultural productivity by 1.5%–24%, placing considerable pressure on food security and economic development in arid regions [7].

With the continued advancement of urbanisation, cities located in arid and semi-arid regions are facing increasingly severe dust-related risks. On the one hand, global climate change has increased the frequency of droughts and intensified extreme weather events. On the other hand, human activities, including land-use change, overgrazing, and urban expansion, have aggravated surface disturbance and further increased the potential for dust emissions. Research indicates that the combined effects of climate change and land-use change are increasing the intensity and frequency of SDS in certain regions [8]. As urbanisation expands in arid and semi-arid regions, desert cities face a compound challenge involving atmospheric degradation, exposed land, and severe water scarcity. Urban greening can contribute to surface stabilisation and environmental improvement, but its performance depends on the spatial arrangement of vegetation, the surrounding built environment, and the water required to sustain planted areas.

Despite substantial progress, three gaps remain. First, existing studies often evaluate vegetation benefits and water demand separately, providing limited quantitative evidence on their spatial coordination within desert cities. Second, many studies report global average relationships and therefore provide insufficient evidence on how the effects of vegetation cover and landscape structure vary across locations and stages of urban development. Third, vegetation expansion is sometimes interpreted as evidence of dust mitigation even when direct particulate-matter or dust-event observations are unavailable. These gaps matter because an intervention that stabilises exposed surfaces may simultaneously increase irrigation demand or restrict local ventilation. Uniform greening targets may therefore conceal spatial trade-offs and misallocate scarce water resources.

This study addresses these gaps by treating mean visibility as an indicator of atmospheric clarity and surface soil moisture as an indicator of near-surface water availability. The analysis combines land-cover structure, vegetation indices, geographical detection, global regression, and local regression to identify the spatial and temporal conditions under which greening resources are associated with different coordination states. Accordingly, the study asks: (1) How did visibility–soil-moisture coordination vary spatially and temporally in Dubai? (2) Which vegetation and landscape-pattern indicators showed the strongest and most stable spatial associations with the coordination index? (3) How can the results inform spatially differentiated greening and water-management strategies under desert conditions?

This study makes four specific contributions. First, it applies a visibility–soil-moisture coordination framework to quantify the spatial relationship between atmospheric clarity and near-surface water availability in a desert city. Second, it identifies stage-dependent changes in the relative importance of vegetation cover and landscape structure rather than assuming that a single indicator dominates throughout the study period. Third, it combines geographical detection, OLS regression, and GWR to distinguish factor interactions, global associations, and spatially varying relationships. Fourth, it translates these findings into differentiated planning strategies for coastal built-up areas, urban expansion fringes, and inland desert areas. These contributions are intended to support exploratory environmental diagnosis and spatial planning; they do not establish causal effects of greening on dust or particulate pollution.

## 2. Research Background

### 2.1. Formation and management of dust storms in desert regions

International research has generally approached sand and dust storms from the perspective of their formation mechanisms, with particular attention to the combined effects of climatic conditions, surface characteristics, and wind regimes. Studies have shown that under conditions of limited precipitation, sparse vegetation cover, and high wind speeds, fine surface particles are more readily entrained by wind and transported into the atmosphere, thereby generating large-scale dust-transport processes. Recent research further suggests that the combined effects of climate change and land-use change have increased the frequency of dust storms in some arid regions, with substantial consequences for urban air quality.

In terms of mitigation measures, ecological restoration is widely regarded as an important means of suppressing dust emissions. Increasing vegetation cover can enhance soil stability and reduce wind erosion, thereby limiting the generation and dispersion of airborne dust. Meanwhile, the establishment of shelterbelts and vegetative barriers along urban peripheries has been shown to modify near-surface wind-field structures, reduce wind speeds, and restrict the transport of particulate matter into urban areas. Recent studies have also begun to examine the long-term capacity of urban forests to reduce atmospheric particulate matter. For example, a study of urban shelter forests found that increasing vegetation indices was significantly associated with declining PM_10_ concentrations in urban areas and, to some extent, with reduced health risks related to air pollution [9].

With the continued progression of global climate change, land degradation, and urbanisation, the impacts of dust activity on urban ecosystems in arid and semi-arid regions have attracted increasing international attention [10]. In recent years, sand and dust storms (SDS) have been recognised as a major environmental issue affecting urban air quality, public health, and ecological security. Studies have demonstrated that SDS not only increase airborne particulate-matter concentrations but also influence climate systems and ecological environments at regional and global scales through atmospheric transport [11]. Against this background, international research has gradually shifted from isolated windbreak and sand-stabilisation projects toward integrated ecological-governance approaches that combine urban greening, ecological restoration, and water-resource management to strengthen the stability of urban ecosystems in arid regions. Extensive research has been conducted in the Middle East, the arid regions of North America, and Australia, particularly on dust-generation mechanisms, ecological protection systems, and water-efficient urban greening. These studies provide important experience for the ecological management of desert cities.

### 2.2. Urban greening and water-resource allocation

Efficient water use has consistently been a central concern in the greening of desert cities. Because arid regions are characterised by scarce precipitation and intense evaporation, urban greening must balance ecological benefits against water consumption. Accordingly, international research has generally emphasised water-efficient landscape design and drought-tolerant planting schemes to reduce the dependence of urban green systems on water resources. In recent years, the concept of xeriscaping has been widely adopted in urban planning in arid regions. By reducing the use of water-intensive lawns, introducing drought-tolerant plant species, and optimising irrigation systems, this approach seeks to minimise the water demand of urban landscapes. Studies have shown that water-efficient green-space systems designed to replace conventional lawn-dominated landscapes can substantially reduce irrigation demand and improve water-use efficiency in urban greening [12]. Meanwhile, the use of drought-tolerant plants in urban greening has received increasing attention. Recent research indicates that increasing the proportion of native and drought-tolerant species can not only reduce irrigation demand but also enhance urban ecosystem stability and biodiversity [13]. Urban vegetation also provides multiple ecosystem services, including air-pollutant capture, carbon sequestration, and regulation of the urban water cycle. However, in water-scarce urban environments, a pronounced trade-off persists between the ecological benefits of green systems and the availability of water resources. Maximising the ecological functions of urban greening under water-saving constraints therefore remains an important research priority [14].

In arid and semi-arid cities, greening configuration is not merely a means of improving landscape quality but also a form of critical ecological infrastructure for strengthening ecological resilience, mitigating sand and dust hazards, and enhancing the human living environment [15]. Existing studies have demonstrated that well-designed urban green systems can reduce airborne particulate-matter concentrations through multiple processes, including foliar interception, surface deposition, attenuation of airflow, and regulation of local microclimates, thereby limiting dust dispersion and reducing residents’ exposure risks [16]. Vegetative barriers, multilayered tree–shrub–grass communities, and continuous green corridors are considered particularly effective in reducing particulate matter and improving air quality along open roads, at residential boundaries, and in protective spaces on the urban periphery [17].

Mechanistically, the regulation of dust and particulate matter by urban greening operates through three principal pathways. First, plant leaves, branches, and aerodynamically rough canopies enhance the interception, adsorption, and deposition of particles, thereby reducing near-surface concentrations of suspended particulate matter. Second, appropriately configured plant communities can modify near-ground wind-field structures, attenuate peak wind speeds, and reduce the potential for dust entrainment from exposed surfaces. Third, through transpiration and shading, green spaces improve local thermal conditions and turbulent exchange, which in turn affect pollutant dispersion and particle resuspension [18]. Previous reviews have confirmed that urban vegetation can facilitate pollutant deposition and retention, although its actual effectiveness depends strongly on plant morphological traits, community structure, spatial configuration, and background meteorological conditions [19].

It should be noted that the capacity of urban greening to reduce particulate matter does not depend simply on increasing the quantity of vegetation but, more importantly, on the suitability of its spatial configuration. Recent studies suggest that urban green spaces generally reduce particulate-matter exposure at the city or district scale. In poorly ventilated environments such as street canyons, however, inappropriate vegetation arrangements may obstruct air exchange and cause local pollutant accumulation. Urban greening should therefore extend beyond the expansion of green-space area and place greater emphasis on vertical vegetation structure, canopy porosity, greenbelt width, boundary continuity, and spatial relationships with roads and buildings. Such considerations are essential for balancing dust reduction, ventilation, and thermal-environment improvement.

The ecological value of greening configuration is particularly pronounced in desert cities. On the one hand, protective greenbelts located along urban peripheries and major sand-transport corridors can reduce near-surface wind speeds, stabilise exposed soils, and suppress wind erosion, thereby limiting the influx of windblown dust. On the other hand, internal urban green-space systems can alleviate the combined effects of air pollution and sand and dust disturbance by improving local microclimates, increasing surface roughness, and enhancing particulate deposition. Urban greening should therefore be understood not only as an amenity serving aesthetic and recreational purposes but also as a functional spatial system for managing sand and dust risks.

### 2.3. Design and application of landscape coupling

In recent years, landscape-coupling design has gradually emerged as an important concept in urban ecological planning for arid regions. This approach emphasises the integrated consideration of natural factors, including climatic conditions, topography, vegetation types, and soil structure, and seeks to enhance the multifunctionality of urban ecosystems through multiscale landscape configuration. Studies have shown that composite landscape systems comprising windbreak greenbelts, ecological corridors, and urban green-space networks can effectively improve the urban ecological environment. For example, the establishment of continuous green infrastructure at the landscape scale can not only strengthen ecological connectivity but also reduce the dispersion of air pollutants and improve the urban microclimate to a certain extent [20].

Some studies have also examined the role of vegetation in pollution control from the perspective of plant functions. Plants can capture airborne particulate matter and gaseous pollutants through foliar deposition and adsorption, thereby reducing ambient pollution levels. This plant-based approach to environmental purification, commonly referred to as phytoremediation, has increasingly been recognised as a sustainable ecological-management strategy [21]. Meanwhile, growing attention has been directed towards the influence of landscape configuration on the ecological performance of urban green systems. Relevant studies indicate that the ecological benefits of urban greening depend not only on vegetation coverage but also on spatial structure, community composition, and management practices.

### 2.4. Research gaps and future directions

Overall, international research has made considerable progress in the management of dust storms and the configuration of urban greening in desert cities. Several limitations, however, remain. First, existing studies have largely focused on individual ecological interventions or local-scale analyses, whereas integrated management approaches at the urban or regional scale remain insufficiently explored. Second, under water-resource constraints, systematic quantitative evidence remains limited regarding how the ecological benefits of urban green systems can be enhanced without substantially increasing municipal water consumption.

As urban environmental problems become increasingly complex, some scholars have proposed reconsidering urban ecological governance from the perspective of the water–energy–food–environment nexus. Because these components are linked through complex interactions, future urban environmental governance requires more integrated and systems-oriented research approaches. Further studies may advance in three principal directions. First, interdisciplinary research should be strengthened by integrating ecology, meteorology, hydrology, urban planning, and related fields to establish a comprehensive framework for urban ecological governance. Second, greater emphasis should be placed on quantitative simulation by combining modelling approaches with long-term monitoring data to evaluate the dust-reduction performance of alternative greening configurations. Third, intelligent monitoring technologies should be developed by integrating remote sensing, big data analytics, and sensor networks to dynamically monitor urban green systems and dust processes, thereby improving the scientific robustness and spatial precision of ecological governance.

## 3. Study area and data sources

### 3.1. Overview of the study area

Dubai is one of the seven emirates of the United Arab Emirates (UAE), located on the southeastern coast of the Persian Gulf in the northeastern Arabian Peninsula, at approximately 25°16′ N and 55°18′ E (Fig 1). It borders the Persian Gulf to the west, Sharjah to the north, Abu Dhabi to the south, and lies close to Oman in the southeast. With a coastline of approximately 72 km, Dubai occupies a strategic position in regional shipping, trade, and urban development. The terrain is generally flat, with most urban areas situated on low-lying coastal plains that gradually transition into the Arabian Desert inland. The natural landscape is dominated by desert and sparse vegetation, resulting in a fragile ecosystem highly sensitive to climate change and human disturbance. The UAE lies within a typical arid to hyper-arid climatic zone characterised by limited rainfall, intense evaporation, and low vegetation cover. These conditions pose substantial challenges for urban planning, ecological management, water-resource allocation, and greening development. Benefiting from its coastal location and port infrastructure, Dubai has developed into a major international centre for trade, tourism, aviation, and logistics. Dubai International Airport is one of the world’s busiest international aviation hubs, connecting Europe, Asia, and Africa. However, rapid urban expansion within a desert environment has intensified pressures associated with extreme aridity, land degradation, windblown sand, and ecological conservation.

**Fig 1:**
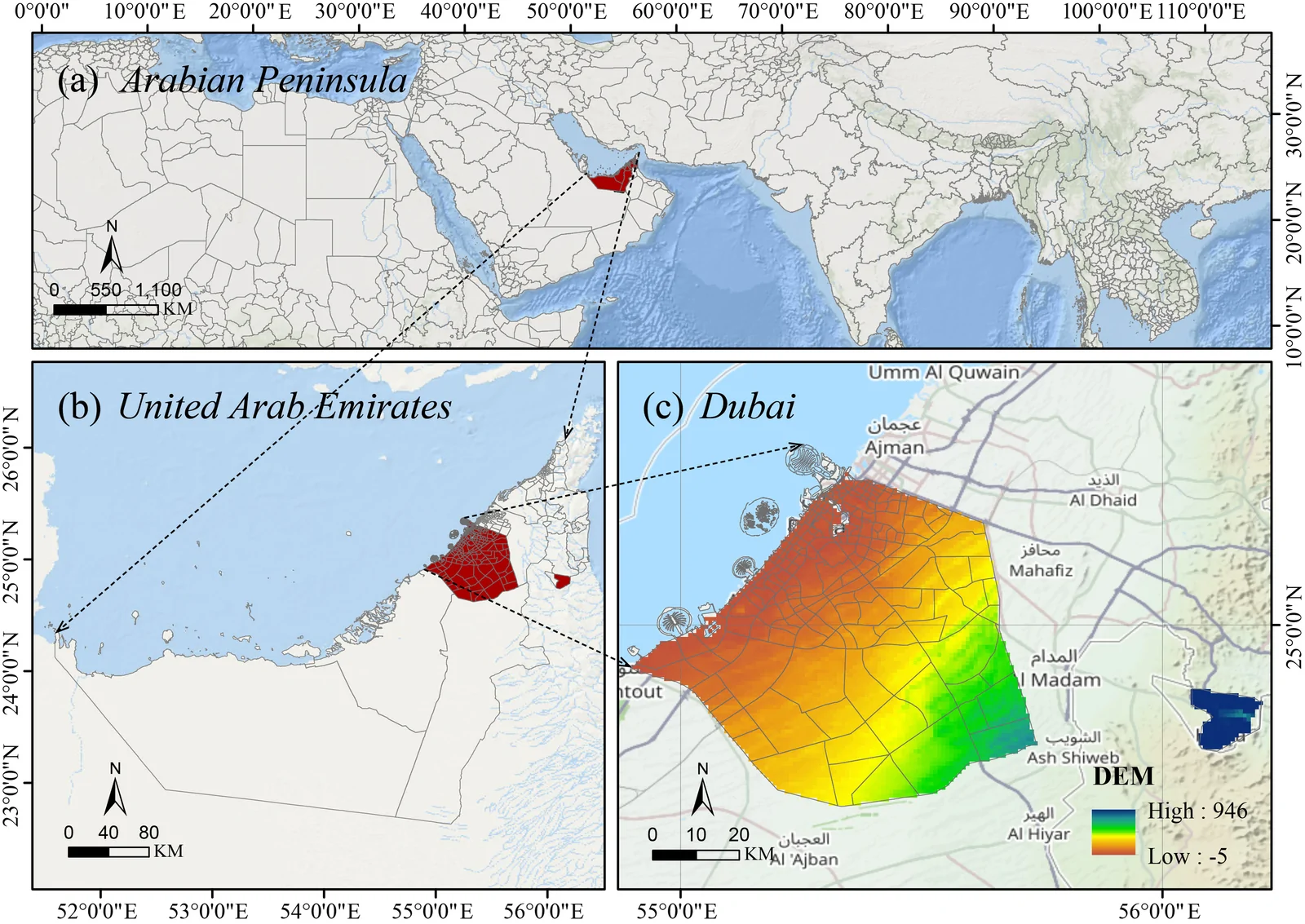
Location of the study area (Boundary data: geoBoundaries, https://www.geoboundaries.org/, CC BY 4.0.).

Dubai has a typical hot-desert climate, characterised by year-round aridity, limited precipitation, extremely hot summers, and mild winters. Annual precipitation is generally below 100 mm and occurs mainly during winter and early spring, often as short-duration rainfall events. Summers, particularly from June to September, are extremely hot, with daytime maximum temperatures frequently exceeding 40 °C and occasionally reaching 45–50 °C (Fig 2). Proximity to the Persian Gulf also results in high summer humidity, further intensifying thermal discomfort. Winters are comparatively mild, with average temperatures of approximately 20–25 °C, making this the principal season for tourism and outdoor activities. The interaction between humid coastal air and the hot desert environment produces pronounced thermal stress. Strong solar radiation, persistent drought, and evaporation rates far exceeding precipitation further aggravate water scarcity and constrain vegetation growth.

**Fig 2:**
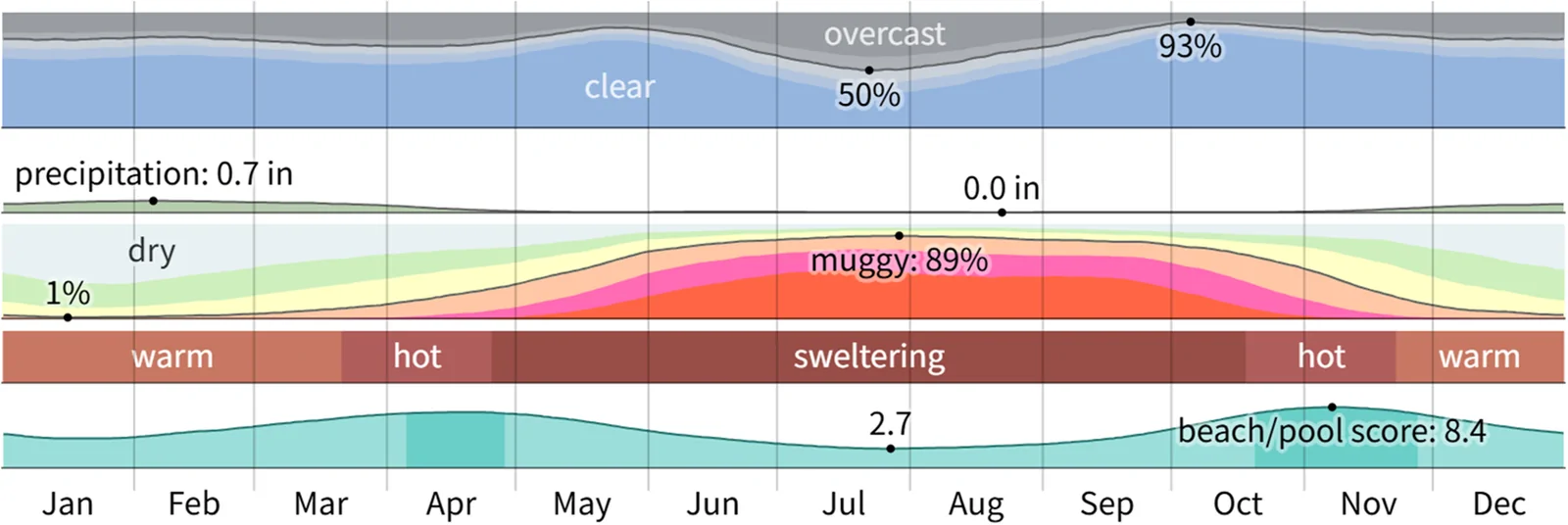
Monthly weather conditions in Dubai. (Source: WeatherSpark, “Average Weather in Dubai, United Arab Emirates, Year Round”).

Dubai has undergone rapid economic transformation over recent decades. Although its early development relied partly on oil production and exports, limited reserves and exposure to oil-price fluctuations prompted the emirate to pursue economic diversification from the late twentieth century onwards. Its economy is now dominated by trade, tourism, aviation, financial services, logistics, real estate, and construction. According to the Dubai Statistics Center, non-oil industries account for more than 90% of Dubai’s gross domestic product. Dubai has also become a major commercial and financial hub in the Middle East. The Dubai International Financial Centre provides regulatory, legal, and financial-service infrastructure for institutions operating across the Middle East, Africa, and South Asia. Large-scale urban developments, including the Burj Khalifa and Palm Jumeirah, have strengthened the city’s global image and supported tourism and real-estate growth. Although the property market experienced a substantial adjustment during the 2008 global financial crisis, it subsequently recovered through policy intervention and renewed international investment. Real estate and construction remain important contributors to Dubai’s economic growth [22].

Dubai is also a highly internationalised city with a predominantly expatriate population. Its resident population exceeds 3.5 million, of whom more than 85% are foreign nationals. Large migrant communities from South Asia, Southeast Asia, Europe, and North America support the construction, service, commercial, and professional sectors. This demographic structure reflects Dubai’s dependence on international labour and capital flows and reinforces its status as a global city [23]. The resulting multicultural environment is evident in the city’s commercial activities, education, entertainment, and lifestyles. Dubai has developed an extensive transport and infrastructure system. Its automated metro network is among the largest in the Middle East, while continued investment in roads and public transport has improved urban accessibility. In recent years, infrastructure development has increasingly incorporated smart-city and sustainability principles, including intelligent transport systems, green buildings, and renewable-energy applications. Substantial investment has also been directed towards green infrastructure and low-carbon urban development [24]. Through green-building regulations and sustainability strategies, the government has promoted energy-efficient technologies and environmentally responsible materials to reduce the environmental pressures associated with rapid urban growth.

### 3.2. Data sources

This study selected 2010, 2015, and 2020 as the reference years and constructed a multisource environmental dataset (Table 1). Vegetation parameters, including the Normalized Difference Vegetation Index (NDVI), Leaf Area Index (LAI), and Fractional Vegetation Cover (FVC), were derived from Landsat imagery at a spatial resolution of 30 m. Land-use information, including bare land, impervious surfaces, and artificial vegetation, was obtained from the GLC_FCS30D dataset. Visibility, precipitation, and surface soil moisture at a depth of 0–7 cm were derived from the Dubai-region subset of the global ERA5 reanalysis dataset. Google Earth Engine was primarily used for remote-sensing image preprocessing, annual compositing, and vegetation-parameter retrieval.

**Table 1:** Summary of data sources.

Data Type	Specific Indicators	Spatial Resolution	Data Source
Remote-sensing vegetation data	NDVI, LAI, and FVC	30 m	Landsat 5/7/8/9 Collection 2 imagery. Cloud removal, annual compositing, and vegetation-parameter retrieval were conducted using the Google Earth Engine (GEE) cloud-computing platform. USGS imagery download: https://earthexplorer.usgs.gov; GEE platform: https://earthengine.google.com
Meteorological data	Visibility and total daily precipitation	0.25°	ERA5 hourly data on single levels from 1940 to present were used to obtain visibility, total precipitation, and volumetric soil water layer 1 (0–7 cm) for the Dubai region in 2010, 2015, and 2020. The dataset was produced by the European Centre for Medium-Range Weather Forecasts (ECMWF) and distributed through the Copernicus Climate Change Service (C3S) Climate Data Store. It provides globally gridded hourly data at a spatial resolution of 0.25° × 0.25°. The hourly variables were aggregated to the daily and annual temporal scales required for the analysis. Official website: https://cds.climate.copernicus.eu/datasets/reanalysis-era5-single-levels?tab=overview; dataset DOI: https://doi.org/10.24381/cds.adbb2d47.
Soil data	Surface soil moisture at a depth of 0–7 cm	0.25°	
Land-use data	Bare Land, Impervious Surface, Managed Vegetation, Sparse Vegetation, Water, and Wetland	30 m	Land-cover maps for 2010, 2015, and 2020 were obtained from the GLC_FCS30D global 30 m land-cover dynamics dataset, which provides annual land-cover maps after 2000. The original land-cover subclasses were reclassified into six categories, namely impervious surface, managed vegetation, sparse vegetation, bare land, water, and wetland, using a consistent crosswalk for all three years. The same spatial mask, reclassification scheme, and raster-processing parameters were applied to ensure temporal comparability.

## 4 Research Methods

### 4.1. Calculation of the air–water coupling coordination degree

Coupling coordination analysis provides an effective means of examining interactions among environmental factors. In desert cities characterised by extreme climatic conditions, the coordination between atmospheric conditions and water availability is particularly important for sustainable urban development. In this study, the air–water coupling coordination degree was assessed through the following three components.

(1) Spatiotemporal variation in mean visibility: Visibility is commonly used as an indirect indicator of atmospheric conditions because it is sensitive to changes in airborne particulate matter, including PM_10_ and PM_2.5_. Analysing visibility across different periods and locations can reveal the spatiotemporal evolution of atmospheric pollution under varying climatic conditions. It also provides a basis for examining the relationships among dust storms, dust-weather events, and urban air quality.
(2) Spatiotemporal variation in soil moisture: Soil moisture is a key indicator of water availability and directly affects vegetation growth, ecosystem functioning, and the environmental regulation capacity of urban green spaces. Its spatiotemporal variation can be used to evaluate the capacity of different greening configurations to retain soil water. In desert cities, changes in soil moisture are closely related to irrigation demand and water-use efficiency, thereby influencing the coordination between atmospheric conditions and water resources.
(3) Coupling coordination analysis: The coupling coordination model integrates atmospheric and water-resource indicators to quantify the interaction and coordination between the two subsystems. By incorporating indicators such as visibility, particulate-matter concentration, and soil moisture, the model evaluates the extent to which air quality and water availability develop in a coordinated manner. The resulting coupling coordination degree can further be used to assess the environmental effects of different urban greening configurations and water-resource management strategies.

### 4.2. Construction of the urban greening resource driver system

Urban green spaces play a critical role in the environmental management of desert cities. Quantitative assessment of greening resources can clarify their contributions to dust-storm mitigation, air-quality improvement, and water-resource regulation. In this study, urban greening resources were characterised using two groups of indicators: landscape-pattern metrics and vegetation indices.

#### 4.2.1. Landscape-pattern metrics.

Landscape-pattern analysis uses remote-sensing imagery and land-use classification to evaluate the spatial distribution and structural characteristics of urban green spaces. Green-space patches were extracted from the classified land-use data and analysed using FRAGSTATS and related spatial-analysis tools. The resulting metrics describe patch composition, fragmentation, geometric complexity, aggregation, and connectivity, providing a quantitative basis for examining how green-space patterns affect air quality, dust control, and water-resource use.

The principal landscape-pattern metrics are described below.

(1) Percentage of Landscape (PLAND): PLAND represents the proportion of the total landscape occupied by a particular green-space patch type. It can be used to assess the relative abundance of different green-space types and their potential contributions to air-quality improvement and microclimate regulation.
(2) Patch Density (PD): Patch density refers to the number of patches of a particular land-cover type per unit area. A higher PD generally indicates a greater degree of landscape subdivision or fragmentation. It is therefore an important indicator of the fragmentation and spatial continuity of urban green spaces.
(3) Edge Density (ED): Edge density represents the total length of patch boundaries per unit landscape area, commonly expressed as metres per hectare. It reflects the extent of edge development and the geometric complexity of green-space patches. Higher ED values generally indicate more complex or fragmented patch boundaries, although their ecological effects depend on the surrounding landscape context.
(4) Landscape Shape Index (LSI): The Landscape Shape Index measures the overall geometric complexity of landscape patches. Higher LSI values indicate increasingly irregular patch shapes and more complex spatial structures. In urban greening, patch shape can influence airflow regulation, ecological interactions, habitat provision, and the exposure of vegetation to surrounding environments. Appropriate shape design may therefore contribute to wind attenuation and dust interception in desert cities.
(5) Shannon’s Diversity Index (SHDI): Shannon’s Diversity Index measures the diversity of land-cover or green-space types within a landscape. Higher values indicate greater compositional diversity and a more balanced distribution among landscape types. In desert cities, a diversified green-space system may enhance ecological adaptability and multifunctionality, particularly when drought-tolerant species and vegetation communities with different ecological functions are appropriately combined.
(6) Aggregation Index (AI): The Aggregation Index measures the spatial concentration of patches belonging to the same landscape type. Higher values indicate that patches are more strongly clustered. Aggregated green spaces can improve habitat continuity, strengthen ecological stability, and facilitate the concentrated use of irrigation resources. In desert cities, an appropriate degree of aggregation may also enhance wind protection, sand stabilisation, and local microclimate regulation.
(7) Proximity Index (PROX): The Proximity Index evaluates the spatial relationships between a focal patch and neighbouring patches of the same type within a specified search distance. Higher values indicate that nearby patches are larger, more numerous, or more closely distributed. A high proximity level may support the formation of ecological corridors and strengthen functional linkages among urban green spaces.
(8) Effective Area Index (EAI): The Effective Area Index characterises the relationship between the usable interior area of a green-space patch and its boundary effects. Higher values generally indicate a larger effective core area and a lower relative influence of patch edges. Green spaces with larger effective areas may provide more stable ecological functions, including pollutant removal, dust interception, and efficient water use.
(9) Landscape Connectivity Index (LCI): The Landscape Connectivity Index measures the degree of spatial or functional connection among green-space patches. Higher values indicate a more continuous and integrated ecological network, which can facilitate species movement, ecological flows, and the maintenance of ecosystem functions. In desert cities, connected green-space networks may form protective corridors, improve dust interception, and enhance the overall environmental performance of urban greening systems.

#### 4.2.2. Vegetation characterisation indices.

Vegetation indices are important indicators of the ecological condition and ecosystem-service capacity of urban green spaces. They facilitate quantitative assessment of the role of urban vegetation in improving air quality and reducing PM_2.5_ and PM_10_ concentrations [25]. The principal vegetation indicators considered in this study are described below.

(1) Normalised2 Vegetation Characterisation Indices: Vegetation indices are important indicators of the ecological condition and ecosystem-service capacity of urban green spaces. They facilitate quantitative assessment of the role of urban vegetation in improving air quality and reducing PM_2.5_ and PM_10_ Difference Vegetation Index (NDVI)

The Normalised Difference Vegetation Index is widely used to characterise vegetation greenness and growth condition. NDVI enables the quantitative assessment of vegetation distribution and activity and can therefore be used to examine the potential effects of different greening types on urban air quality. In desert cities, it is particularly useful for identifying spatial variations in vegetation condition and evaluating the ecological performance of irrigated green spaces.

(2) Fractional Vegetation Cover (FVC): Fractional Vegetation Cover represents the proportion of the ground surface covered by vegetation. Higher FVC generally indicates greater vegetation continuity and a stronger capacity for soil stabilisation, erosion control, and microclimate regulation. In desert cities, increased FVC may reduce exposed soil surfaces, suppress wind erosion, and contribute to lower concentrations of windblown PM_10_ and PM_2.5_.
(3) Leaf Area Index (LAI): The Leaf Area Index is defined as the one-sided leaf area per unit ground area and is a key indicator of vegetation canopy structure. Higher LAI values generally indicate denser foliage and a larger leaf surface available for photosynthesis, evapotranspiration, carbon uptake, and pollutant deposition. The desert cities, LAI provides important information on vegetation growth and ecological functioning under extreme climatic and water-resource constraints. It can be estimated using remote sensing, field measurements, and vegetation-growth models. LAI data can support urban greening design and water-resource management by identifying areas with insufficient canopy development or excessive irrigation demand. Appropriate enhancement of LAI may improve carbon sequestration, thermal regulation, and particulate-matter interception, although its effects depend on plant species, canopy porosity, irrigation conditions, and local airflow.
(4) Vegetation Height: Vegetation height is a basic indicator of plant growth and the vertical structure of urban green spaces. It influences airflow, shading, roughness, and the interception of airborne particles. Taller vegetation, particularly trees and multilayered plant communities, can attenuate wind speeds and intercept windblown dust. However, its actual effect on pollutant dispersion depends on planting density, canopy permeability, surrounding buildings, and local ventilation conditions.
(5) Vegetation Biomass: Vegetation biomass represents the total quantity of plant material within a green space or study area, including above-ground and below-ground components such as roots, stems, branches, and leaves. It reflects the capacity of vegetation to provide ecosystem services, including carbon sequestration, microclimate regulation, soil stabilisation, and pollutant interception. Biomass can be estimated by combining remote-sensing data, field sampling, and vegetation-growth models. Monitoring vegetation biomass is particularly important in desert cities because plant growth is strongly constrained by water availability, temperature, and soil conditions. Temporal changes in biomass can therefore indicate the ecological adaptability and resource-use efficiency of urban vegetation under different climatic conditions. Increasing biomass through appropriately diversified and water-efficient planting may strengthen wind and sand control, thermal regulation, and pollutant-removal capacity.
(6) Vegetation Morphology: Vegetation morphology refers to external structural traits such as plant height, crown width, branching form, leaf size, and leaf shape. These characteristics affect the interactions between vegetation and its surrounding environment and consequently influence shading, airflow regulation, particle capture, and water consumption. The assessment of vegetation morphology is especially relevant in desert cities, where plant form is closely associated with climatic adaptation, soil conditions, and irrigation availability. Drought-tolerant species commonly possess smaller leaves, specialised canopy structures, or extensive root systems that improve their ability to withstand water stress. Trees with broad crowns can provide substantial shade and alleviate urban heat, whereas taller or multilayered vegetation may serve as windbreaks and intercept airborne dust. Morphological analysis therefore provides an important basis for selecting plant species and optimising planting structures.
(7) Green View Index (GVI): The Green View Index measures the proportion of visible vegetation within streets or public spaces and is commonly calculated from street-view imagery. Unlike remotely sensed indicators that primarily describe vegetation from an overhead perspective, GVI reflects greenery as directly perceived by pedestrians. It is therefore relevant to the assessment of environmental quality, visual comfort, psychological well-being, and urban liveability. GVI is usually calculated by identifying vegetation pixels in street-view images using image-processing or computer-vision techniques. In desert cities, where the expansion of green-space area is constrained by limited land and water resources, improving visible greenery through strategic spatial design may offer a resource-efficient means of enhancing the urban environment. Quantitative comparisons of GVI among neighbourhoods can identify disparities in pedestrian-level greening and support the optimisation of vegetation location, vertical structure, and visual exposure in densely used urban areas.

### 4.3. Global and local models of driving mechanisms

To investigate the relationship between landscape patterns and the air–water coupling coordination degree, this study developed global and local driving models to quantify the effects of landscape-pattern characteristics on atmospheric conditions and water availability. A geographically weighted regression model and the optimal-parameter geographical detector (GeoDetector) were employed to identify the overall and spatially differentiated effects of landscape-pattern changes on urban ecological functions, thereby providing a quantitative basis for urban greening planning and water-resource management.

The global analysis focused on the overall relationships between landscape-pattern metrics and the air–water coupling coordination degree. XGBoost was used to assess the combined effects of indicators such as percentage of landscape, patch density, and edge density on atmospheric and water-resource conditions, including particulate-matter concentrations, soil moisture, and irrigation demand. This approach identifies the relative importance and nonlinear effects of landscape characteristics across the entire study area, thereby clarifying the overall environmental performance of alternative green-space configurations.

The local analysis examined the spatial heterogeneity of these relationships. In desert cities, differences in climate, soil, vegetation, and urban development may cause the effects of landscape patterns to vary substantially among locations. The geographically weighted model was therefore used to estimate spatially varying relationships, while GeoDetector was applied to identify the explanatory power and interactive effects of individual driving factors. These analyses reveal where particular landscape characteristics exert stronger influences on air–water coupling coordination. For example, improving green-space connectivity or optimising patch distribution may produce greater benefits in certain areas by enhancing dust interception, air-quality regulation, and soil-water retention.

#### 4.3.1. Geographically weighted regression model.

The geographically weighted Regression model integrates spatial weighting with the Regression machine-learning algorithm, enabling the simultaneous assessment of global nonlinear relationships and local spatial variations. By assigning distance-based weights to observations, the model accounts for geographical differences in the effects of landscape-pattern characteristics on the air–water coupling coordination degree. The bandwidth selected through spatial block cross-validation was used for the final GWR estimation in each year.

The model was used to identify the relationships between landscape characteristics, including green-space types, patch distribution, and landscape connectivity, and environmental conditions such as air quality and water-resource use. It also quantified the relative contribution of individual landscape features across different locations. The results provide a quantitative basis for optimising green-space configuration, controlling dust hazards, and improving water-resource management.

To evaluate the generalisability of the GWR models, five-fold spatial block cross-validation was conducted separately for 2010, 2015, and 2020. Spatially adjacent observations were assigned to the same block to reduce information leakage caused by spatial dependence. Within each training fold, adaptive neighbourhood sizes of 50, 100, 150, 200, 250, and 300 observations were evaluated, and the bandwidth with the lowest validation error was selected. The model was then refitted using the training observations only and used to predict the observations in the held-out spatial block. Predictive performance was assessed using the cross-validated coefficient of determination, root mean square error, and mean absolute error. The cross-validated R² was calculated from held-out predictions rather than from the fitted values of the training data. Thus, the reported global R² and the spatial cross-validated R² represent sample-fitting performance and spatial out-of-sample performance, respectively.

#### 4.3.2. Optimal-parameter geographical detector.

The optimal-parameter geographical detector was employed to examine the spatial associations between landscape-pattern characteristics and the air–water coupling coordination degree. Its principal advantage lies in its capacity to quantify the explanatory power of individual factors and identify interactions among multiple drivers without requiring a predefined linear relationship.

Landscape metrics, including percentage of landscape, patch density, and edge density, were discretised using optimised classification parameters before factor and interaction detection. This approach enabled the identification of the landscape characteristics that most strongly explained spatial variations in coupling coordination. It also revealed whether interactions between factors produced enhanced, nonlinear, or weakened explanatory effects. The results can support spatially differentiated greening strategies; for example, improving green-space connectivity or landscape diversity may enhance air-quality regulation and water-use efficiency in specific areas.

### 4.4. Validation through small-sample field surveys

To assess the reliability and practical relevance of the large-scale analysis, this study combined multisource big-data analysis with small-sample field surveys. The city-scale assessment integrated remote-sensing imagery, environmental monitoring data, and landscape-pattern metrics to evaluate urban greening and environmental conditions. Although these analyses provided an overall basis for greening planning and environmental optimisation, field validation was necessary because climatic, geographical, and socioeconomic conditions vary considerably across urban areas.

Representative small-scale sites were therefore selected for field observation and data collection. The surveys were used to examine whether the spatial patterns identified through large-scale datasets were consistent with actual site conditions. This process also supported the formulation of site-specific landscape-design strategies and improved the practical applicability of the analytical results.

#### 4.4.1. Framework integrating big-data analysis and small-sample surveys.

The study first conducted a city-scale assessment incorporating green-space distribution, climatic conditions, atmospheric conditions, and water-resource availability. Remote-sensing and geographic information system data were used to identify landscape characteristics associated with environmental quality and ecological balance and to assess their contributions to the air–water coupling coordination degree. These results provided a preliminary basis for optimising urban greening and water-resource management.

Representative locations were subsequently selected for small-scale field surveys. Site observations and environmental measurements were used to collect detailed information on landscape patterns, atmospheric conditions, soil moisture, and microclimatic characteristics. The field evidence was then compared with the large-scale analytical results to evaluate their consistency and reliability.

#### 4.4.2. Site selection and data collection.

Survey sites were selected from key areas identified through the large-scale spatial analysis. To capture differences in geographical and environmental conditions, the sample included urban core areas, peripheral expansion zones, sparsely vegetated areas, and locations with relatively high exposure to windblown dust. All the locations involved in our work are open to the public and do not fall under any restricted or protected areas

Field data collection included the following components:

(1) Air-quality monitoring: Portable monitoring instruments were used to measure PM_2.5_ and PM_10_ concentrations. The observed values were compared with the spatial patterns estimated from the large-scale environmental datasets.
(2) Soil moisture and water-resource use: Soil-moisture sensors and relevant water-use records were used to assess soil-water conditions and the irrigation efficiency of different green-space types.
(3) Landscape patterns and green-space distribution: Field observations and GIS-based spatial analysis were combined to document the location, form, composition, and connectivity of green-space patches.
(4) Microclimatic monitoring: Temperature, relative humidity, and wind speed were measured using portable environmental instruments to examine the influence of landscape configuration on local microclimatic conditions.

#### 4.4.3. Comparison between field-survey and big-data results.

Following the field surveys, site-level observations were compared with the results of the large-scale spatial analysis. This comparison was used to identify potential deviations caused by data resolution, model assumptions, or local environmental conditions and to refine the interpretation of the analytical results.

Field observations also revealed fine-scale environmental variations that were difficult to capture using city-scale datasets. For example, inappropriate planting density, discontinuous green-space configuration, or inefficient irrigation could result in locally poor atmospheric conditions or low water-use efficiency. The surveys therefore provided evidence for assessing the applicability of the proposed greening strategies. By linking large-scale spatial patterns with site-specific conditions, the validation process strengthened the practical basis for landscape design and environmental management.

## 5. Results

### 5.1. Analysis of the air–water coupling coordination degree

#### 5.1.1. Spatiotemporal evolution of mean visibility.

Fig 3 shows that mean visibility in Dubai exhibited a clear spatial reorganisation between 2010 and 2020. In 2010, relatively high visibility covered most of the study area. High-value areas extended continuously from the southwestern coast towards the south-central inland region, whereas moderate- and low-value areas were mainly concentrated along the northern coast and in several urban-development zones. By 2015, moderate- and low-visibility areas had expanded across the built-up areas along the Persian Gulf coast and the central urban region. At the same time, high-visibility areas contracted to the southwestern coast and several locally distributed areas in the north. The 2020 pattern remained broadly similar to that of 2015, with low and moderately low values concentrated in the northern coastal urban belt and central region, while relatively high values persisted in the southwestern coastal area and the southeastern desert interior.

**Fig 3:**
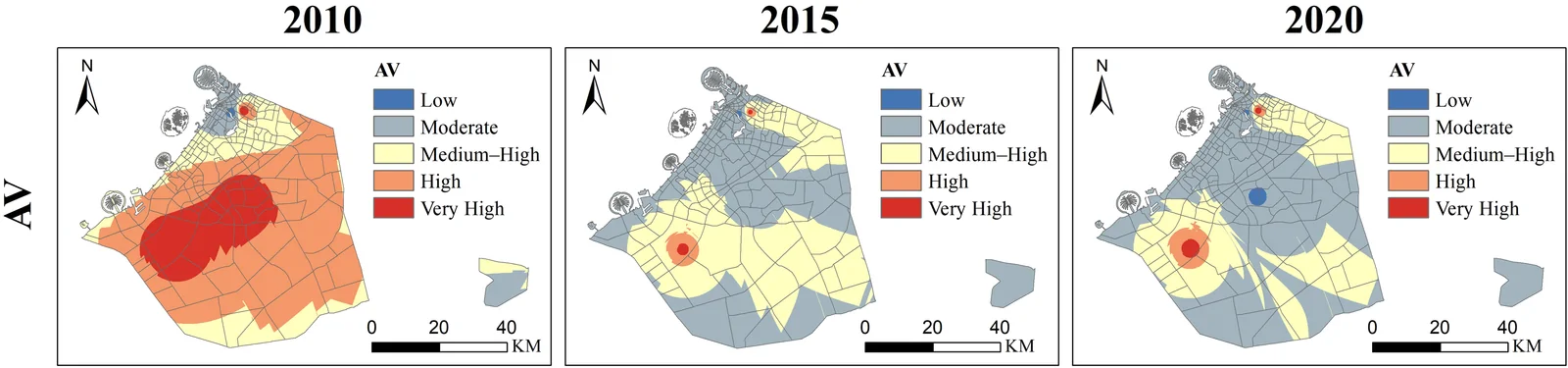
Spatiotemporal evolution of mean visibility (Boundary data: geoBoundaries, https://www.geoboundaries.org/, CC BY 4.0.).

These results indicate that visibility gradually changed from a relatively homogeneous high-value pattern to a more differentiated coastal–inland structure. Lower visibility was concentrated in intensively developed coastal and central urban areas, whereas higher visibility was generally observed in peripheral desert areas. This spatial contrast is compatible with the combined influence of urban development intensity, local atmospheric emissions, maritime humidity, surface disturbance, and the openness of the surrounding desert landscape. The persistence of lower visibility in the northern coastal urban belt also suggests that the deterioration of atmospheric clarity was spatially associated with the main urban-development corridor rather than being distributed uniformly across Dubai. However, because visibility is affected by dust, humidity, precipitation, atmospheric particles, and meteorological conditions simultaneously, the observed pattern should be interpreted as an overall atmospheric-clarity signal rather than as a direct measure of dust concentration alone.

#### 5.1.2. Spatiotemporal evolution of soil moisture.

The spatial distribution of surface soil moisture also changed substantially over the study period (Fig 4). In 2010, soil moisture ranged from 0.049 to 0.089. Higher values were concentrated in the western and southwestern coastal areas and gradually declined towards the central and eastern inland regions, forming a pronounced coast-to-desert moisture gradient. In 2015, the overall range decreased to 0.027–0.056. High-value areas became more localised in the northern and northeastern parts of the study area, while broad low-value zones emerged in the south-central and southwestern inland regions. This pattern indicates that the middle study period was characterised by a widespread reduction in near-surface moisture and a stronger spatial contrast between relatively moist urban or coastal areas and dry inland areas.

**Fig 4:**
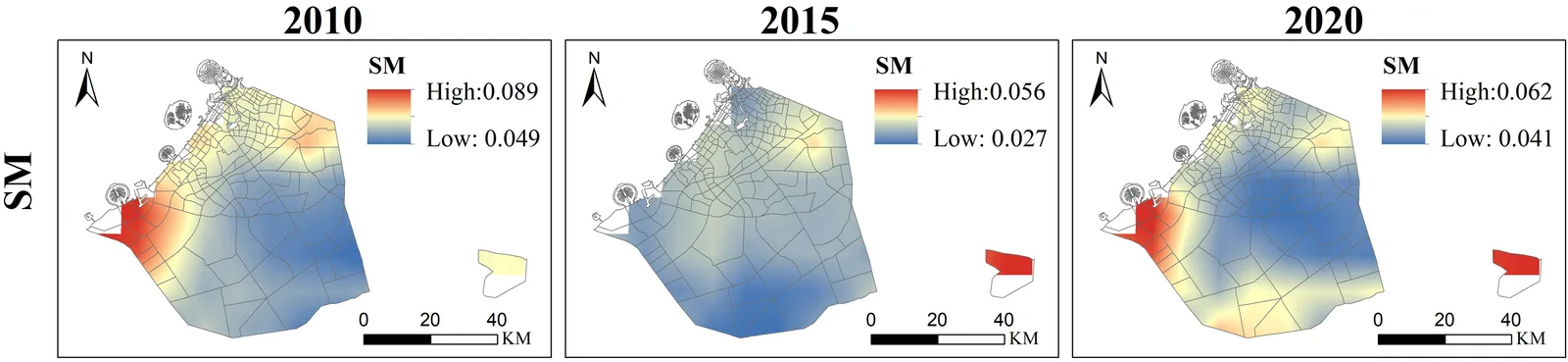
Spatiotemporal evolution of soil moisture (Boundary data: geoBoundaries, https://www.geoboundaries.org/, CC BY 4.0.).

By 2020, soil moisture recovered to a range of 0.041–0.062, and a distinct high-value centre reappeared along the western coast. Nevertheless, the central urban area and eastern inland region remained relatively dry. The recovery was therefore spatially uneven and did not restore the earlier moisture configuration across the entire study area. The Hatta exclave exhibited comparatively high soil moisture in both 2015 and 2020, which differs from the main desert plain of Dubai. This difference is consistent with the distinct topographic, geological, and hydrological conditions of Hatta. The results suggest that the temporal variation in soil moisture was shaped by both broad regional conditions and local landscape settings. In particular, the persistence of dry conditions in the central and eastern regions indicates that the expansion of urban greening alone may not guarantee a corresponding improvement in near-surface water availability.

#### 5.1.3. Coupling coordination analysis.

Fig 5 presents the spatial distribution of the air–water coupling coordination degree. In 2010, moderate and high coordination values dominated Dubai. High-value areas were mainly distributed along the western and southwestern coast, while the central and northern urban areas also maintained relatively favourable coordination levels. Low-value areas were concentrated primarily along the southeastern desert fringe. In 2015, the low-coordination centre shifted towards the southern inland region. At the same time, relatively continuous high-value areas appeared in the northern coastal built-up zone and the northeastern region. Accordingly, the dominant spatial gradient changed from a west–east pattern in 2010 to a more pronounced north–south differentiation in 2015.

**Fig 5:**
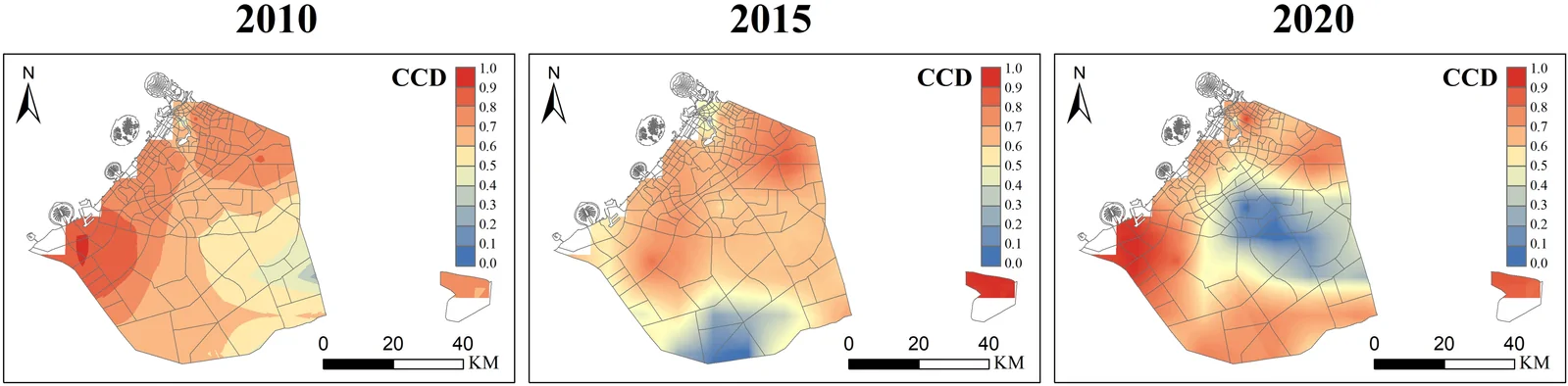
Spatial distribution of the air–water coupling coordination degree (Boundary data: geoBoundaries, https://www.geoboundaries.org/, CC BY 4.0.).

In 2020, moderate and high coordination values remained prevalent in many peripheral areas, but a distinct low-value core emerged in the central urban region and extended towards the east and south. The spatial location of the low-coordination centre therefore shifted successively from the southeastern desert fringe to the southern inland area and then towards the central urban region. This result demonstrates that the air–water coordination state did not follow a simple linear temporal trend. Instead, the spatial focus of environmental constraint changed with the reorganisation of visibility, soil moisture, land use, and urban development.

The coordination index should be interpreted as a measure of the degree of balance between atmospheric clarity and near-surface water availability. A high value does not necessarily mean that both subsystems reached their maximum levels; it indicates that their spatial states were relatively compatible. Conversely, a low value may result from poor visibility, insufficient soil moisture, or a mismatch between the two subsystems. The emergence of low values in the central urban area in 2020 is therefore likely related to the simultaneous presence of relatively poor atmospheric conditions and persistent surface dryness. The migration of low-coordination areas further indicates that environmental management priorities cannot be determined solely by the amount of green space. They must also account for the spatial relationship between atmospheric conditions, water availability, and the surrounding urban landscape.

### 5.2. Urban greening indicators

#### 5.2.1. Landscape-pattern metrics.

The six land-use classes exhibited substantially different spatial configurations and temporal trajectories. These differences indicate that changes in the quantity of green space were accompanied by changes in patch structure, boundary complexity, and spatial continuity. Bare land was the dominant land-use type in Dubai. As shown in Fig 6, high edge-density and patch-density values were concentrated mainly along the northern coastal development fringe, around transport corridors, and at the interfaces between built-up areas and the inland desert. By contrast, the south-central desert interior contained extensive and relatively continuous bare land, which produced lower local edge complexity despite its large total area. Between 2010 and 2020, the spatial framework of high-value bare-land areas remained broadly stable, but local patches became increasingly subdivided and more strongly aligned with urban expansion boundaries and road networks. The reduction in bare-land area therefore occurred together with greater fragmentation of the remaining patches. This indicates that the contraction of the desert matrix was spatially selective, with the most pronounced structural changes occurring at urban–desert transition zones.

**Fig 6:**
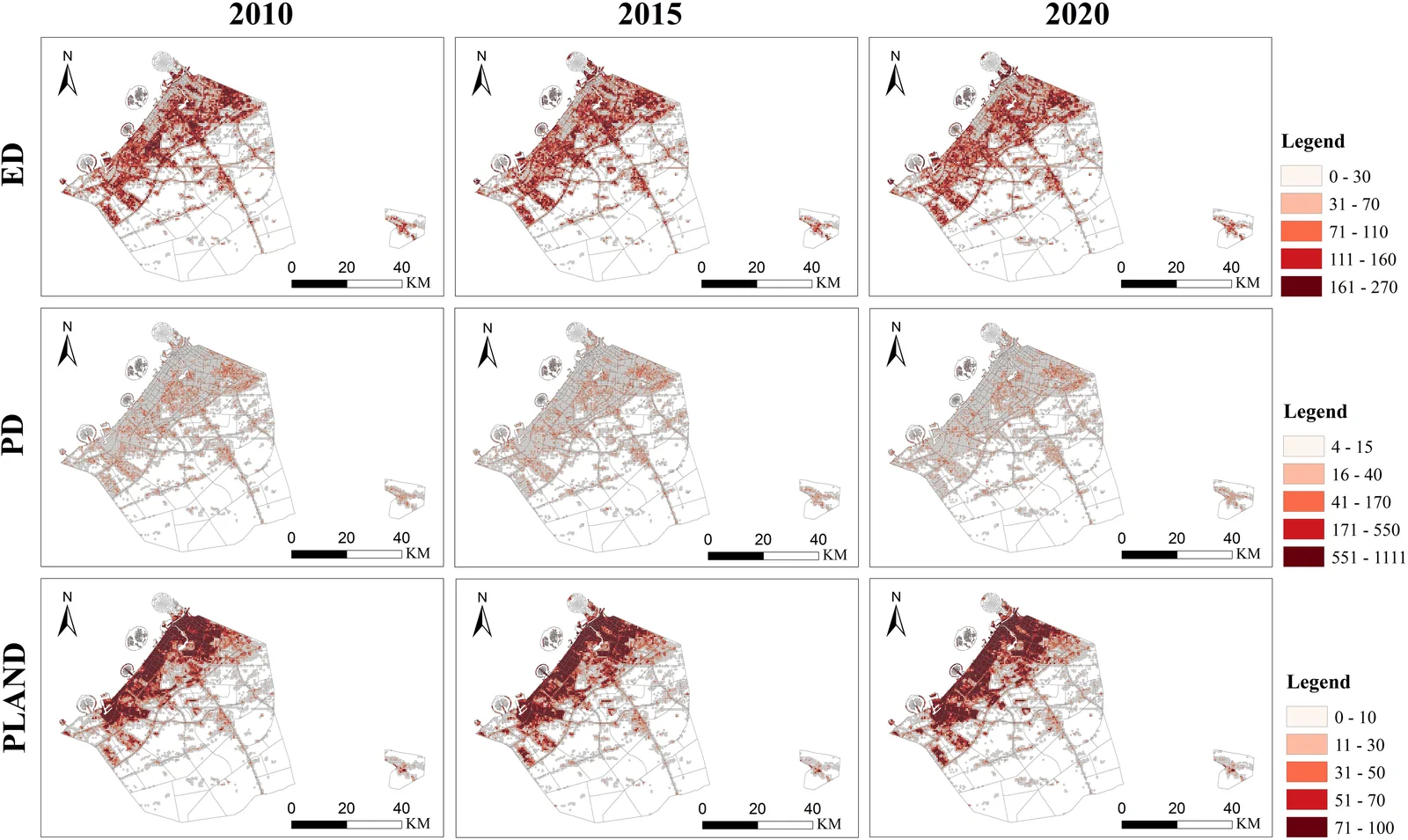
Spatial distribution of landscape-pattern metrics for bare land (C1) (Boundary data: geoBoundaries, https://www.geoboundaries.org/, CC BY 4.0.).

Impervious surfaces were concentrated along the northern and northwestern Persian Gulf coast and extended inland along major transport corridors (Fig 7). This distribution reflects the combined effects of coastal urban concentration and corridor-based expansion. High edge-density values occurred along development fronts, built-up boundaries, and linear infrastructure, whereas relatively high patch density was observed in the northern old city and coastal areas with dense road networks. From 2010 to 2020, the proportion of impervious surfaces increased from 15.27% to 16.99%. However, edge density declined from 34.52 to 33.66 m/ha, and patch density declined from 3.61 to 3.18 patches/100 ha. The simultaneous expansion of impervious surfaces and decline in their edge and patch densities indicates that urban development was accompanied by a degree of spatial consolidation. In other words, built-up land expanded outward while some previously separated urban patches became more spatially connected or integrated into larger developed areas.

**Fig 7:**
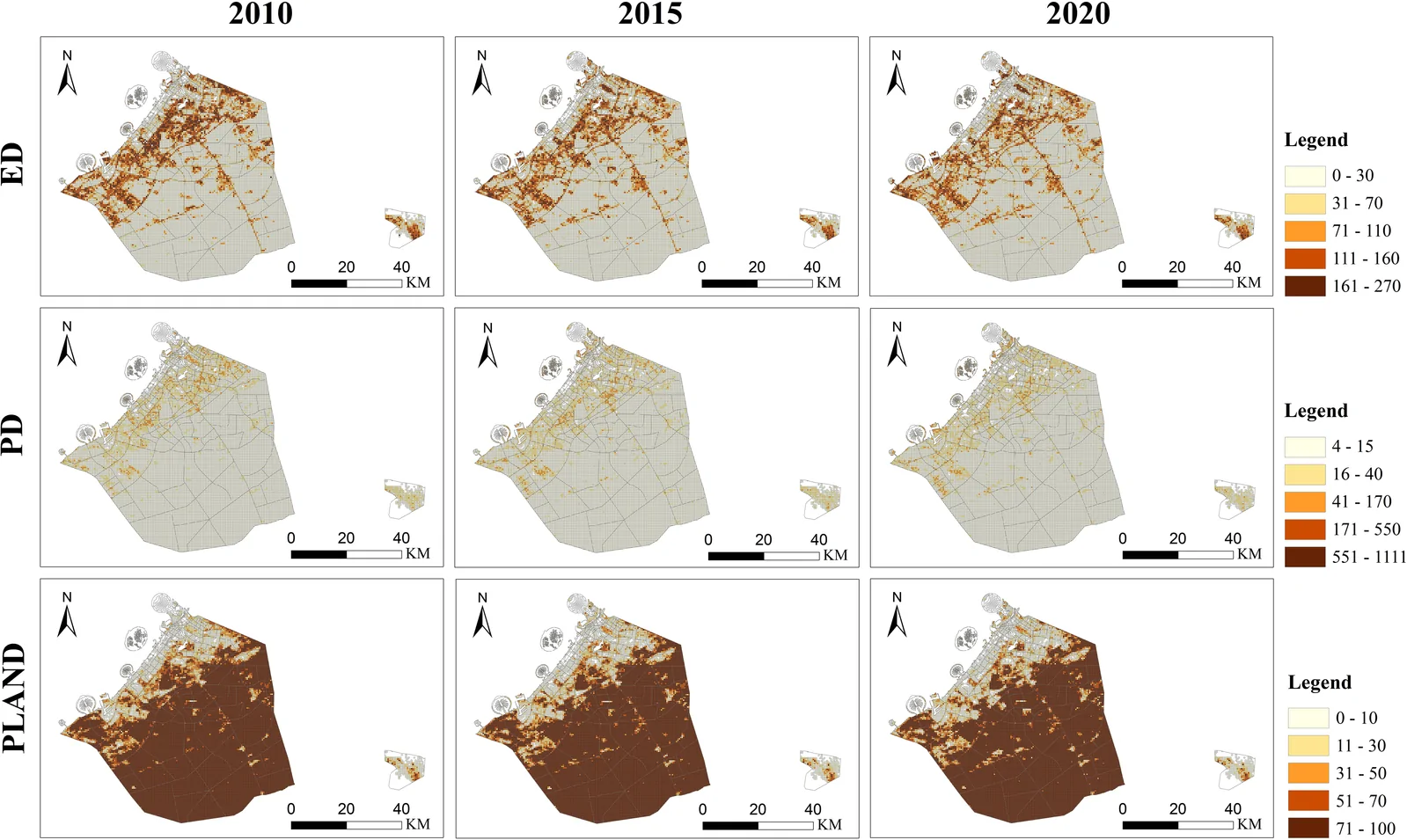
Spatial distribution of landscape-pattern metrics for impervious surfaces (C2) (Boundary data: geoBoundaries, https://www.geoboundaries.org/, CC BY 4.0.).

Managed vegetation remained concentrated in the northern coastal built-up area, residential and public-service spaces, roadside greenbelts, and several inland development nodes (Fig 8). It did not form a continuous citywide network. Instead, it occurred as small patches, linear corridors, and isolated green spaces embedded within the built-up landscape. Its percentage of landscape increased from 3.30% in 2010 to 4.86% in 2020. During the same period, edge density increased from 14.37 to 21.18 m/ha, and patch density increased from 2.85 to 4.10 patches/100 ha. The increase in all three metrics demonstrates that the expansion of managed vegetation was accompanied by increasing boundary complexity and patch subdivision. Therefore, the growth of urban greening was expressed primarily through dispersed parks, roadside planting, residential green spaces, and small development-related patches rather than through the formation of a continuous ecological network. This distinction is important because a larger green-space area does not automatically indicate stronger connectivity, greater ecological continuity, or more efficient use of irrigation water.

**Fig 8:**
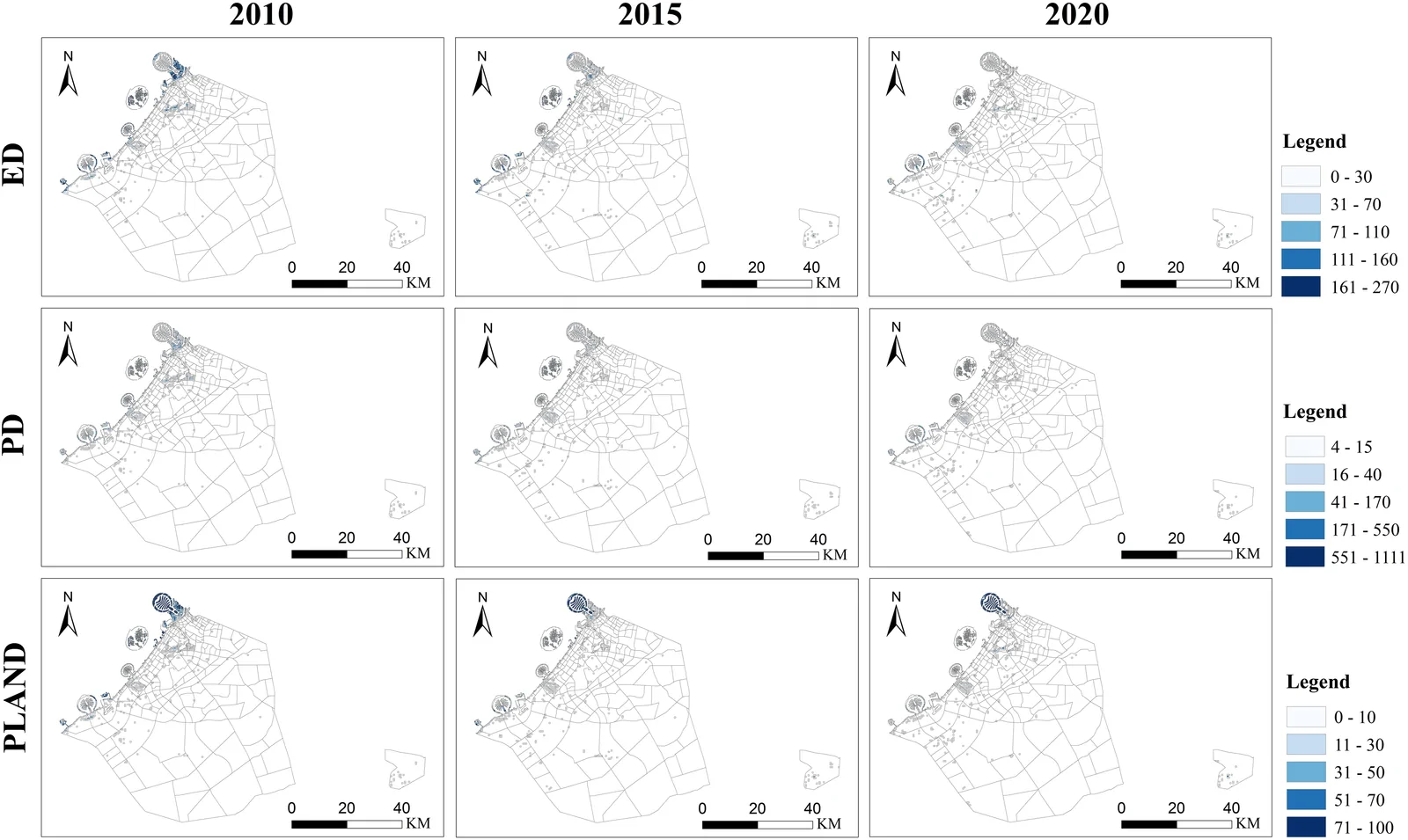
Spatial distribution of landscape-pattern metrics for managed vegetation (C3) (Boundary data: geoBoundaries, https://www.geoboundaries.org/, CC BY 4.0.).

Sparse vegetation displayed a more stable and semi-natural spatial pattern (Fig 9). It was mainly distributed along the outer northern coastal urban area, at the interfaces between built-up land and bare land, and along local desert margins. A relatively concentrated distribution also occurred in the Hatta exclave. Compared with managed vegetation, sparse vegetation consisted of more numerous but generally smaller patches and showed weaker spatial continuity. From 2010 to 2020, its edge density increased from 14.26 to 15.58 m/ha, patch density increased from 3.45 to 3.91 patches/100 ha, and its percentage of landscape increased only slightly from 5.24% to 5.40%. These limited changes suggest that natural or semi-natural vegetation experienced only modest spatial expansion. The overall greening transition in Dubai was therefore driven mainly by managed vegetation rather than by the recovery or expansion of sparse natural vegetation.

**Fig 9:**
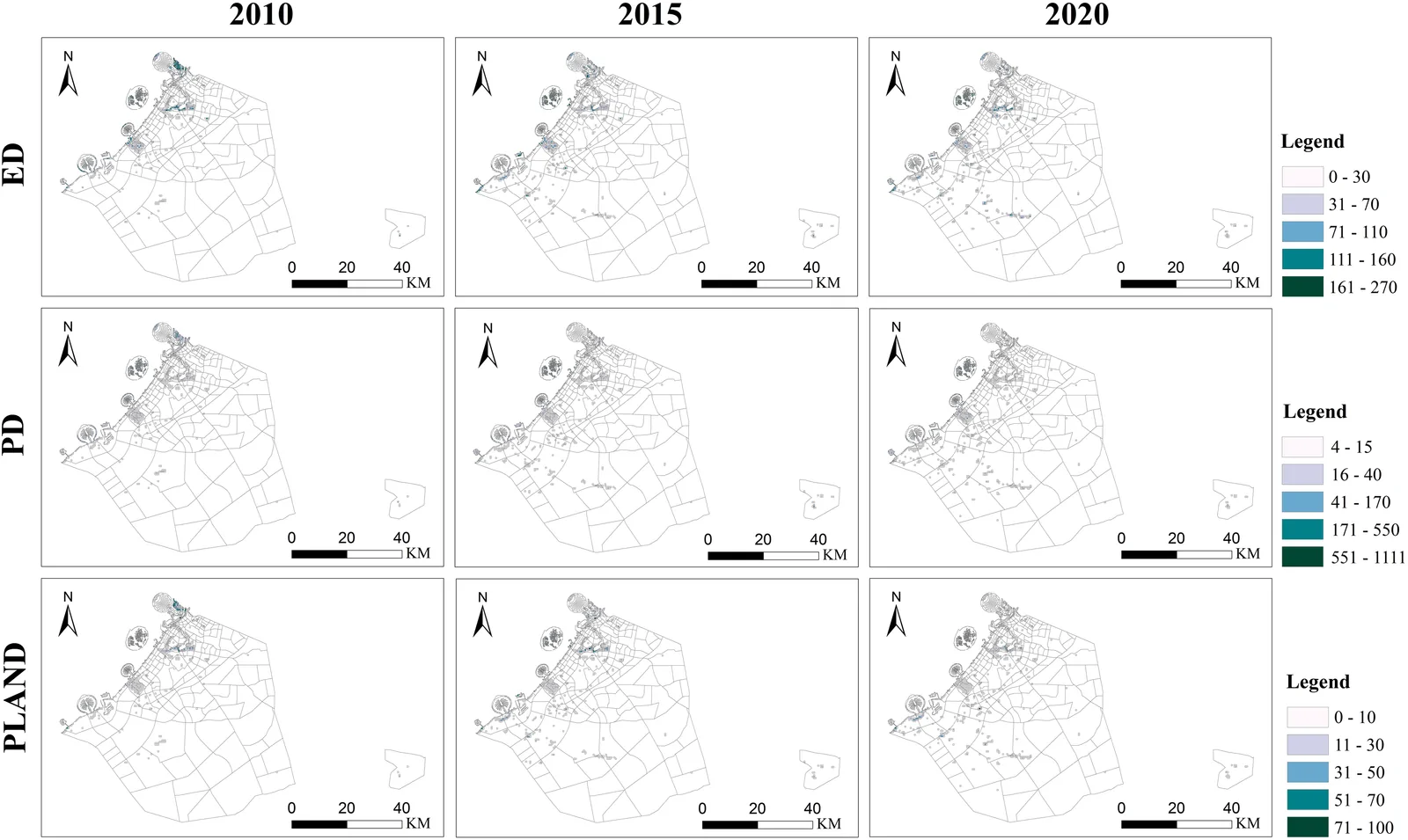
Spatial distribution of landscape-pattern metrics for sparse vegetation (C4) (Boundary data: geoBoundaries, https://www.geoboundaries.org/, CC BY 4.0.).

Water bodies were concentrated along the Persian Gulf coastline, around offshore artificial islands and harbours, and within several internal urban water systems (Fig 10). Their spatial configuration combined a coastal linear structure with discrete artificial-water nodes. High edge-density values occurred mainly along the northern coast, where irregular shorelines, artificial islands, and frequent land–water interfaces generated complex boundaries. Between 2010 and 2020, water-body edge density declined from 2.38 to 1.03 m/ha, patch density declined from 0.55 to 0.50 patches/100 ha, and the percentage of landscape declined from 1.31% to 0.77%. The main locations of water patches remained relatively stable, but their extent and boundary complexity decreased. Water patches in Hatta remained isolated from the coastal water system and exhibited a different spatial form, confirming the functional and geographical separation between the main Dubai territory and the exclave.

**Fig 10:**
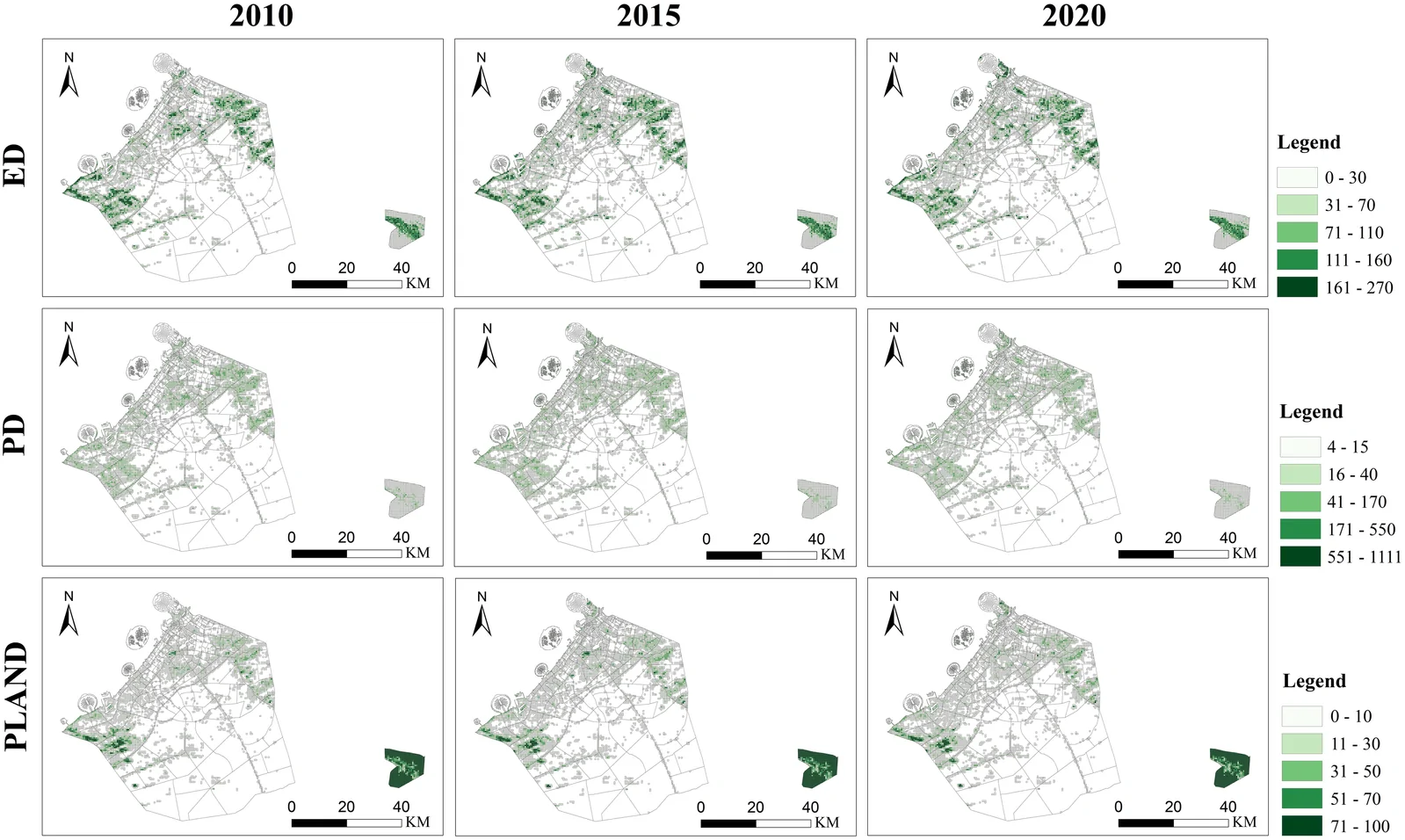
Spatial distribution of landscape-pattern metrics for water bodies (C5) (Boundary data: geoBoundaries, https://www.geoboundaries.org/, CC BY 4.0.).

Wetlands were mainly distributed in low-lying northern coastal areas, around urban waterways, and within artificial waterfront spaces (Fig 11). They formed a combined point–line pattern associated with built-up areas and transport networks. Compared with open water, wetlands extended further into the urban interior but generally consisted of smaller patches with more complex boundaries. Their percentage of landscape declined slightly from 0.37% to 0.34%, while edge density declined from 1.84 to 1.73 m/ha and patch density changed from 0.67 to 0.64 patches/100 ha. The relatively small net changes, together with local expansion and contraction, indicate that the overall wetland framework remained stable but experienced local spatial reorganisation.

**Fig 11:**
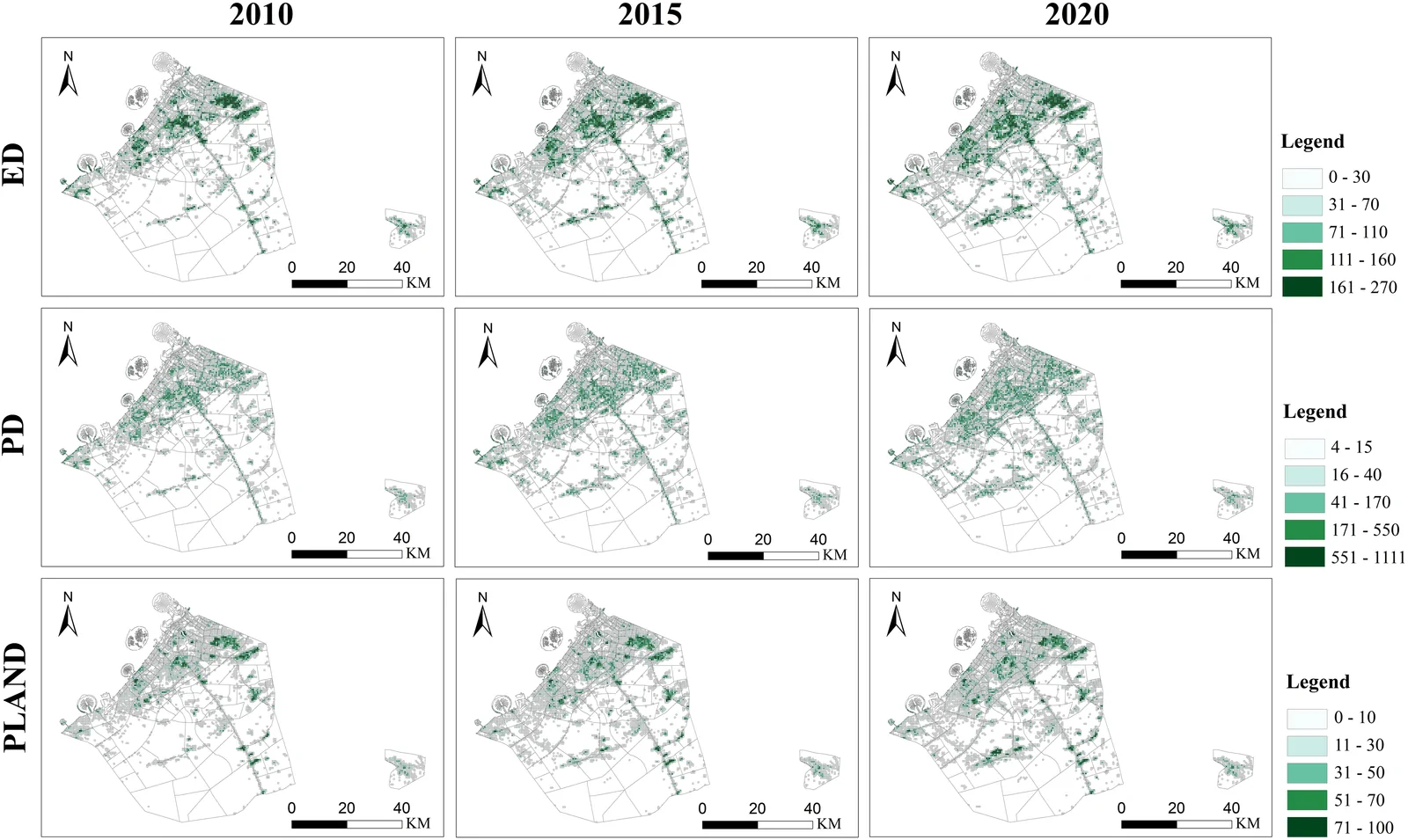
Spatial distribution of landscape-pattern metrics for wetlands (C6) (Boundary data: geoBoundaries, https://www.geoboundaries.org/, CC BY 4.0.).

#### 5.2.2. Vegetation indices.

The spatial distributions of NDVI, FVC, and LAI revealed distinct dimensions of vegetation condition (Fig 12). NDVI and FVC displayed broadly similar patterns. Both indices were generally low in the densely built-up areas along the northern Persian Gulf coast, whereas relatively continuous moderate- and high-value areas were observed in the central-eastern and southeastern inland regions. The maximum NDVI increased from 0.42 in 2010 to 0.46 in 2015 and then declined slightly to 0.44 in 2020. Its minimum value decreased from −0.24 to −0.28 before recovering to −0.21. FVC ranged from 0 to 1 in all three years, although the locations of high- and low-value areas shifted between the northern urban periphery and the south-central inland region.

**Fig 12:**
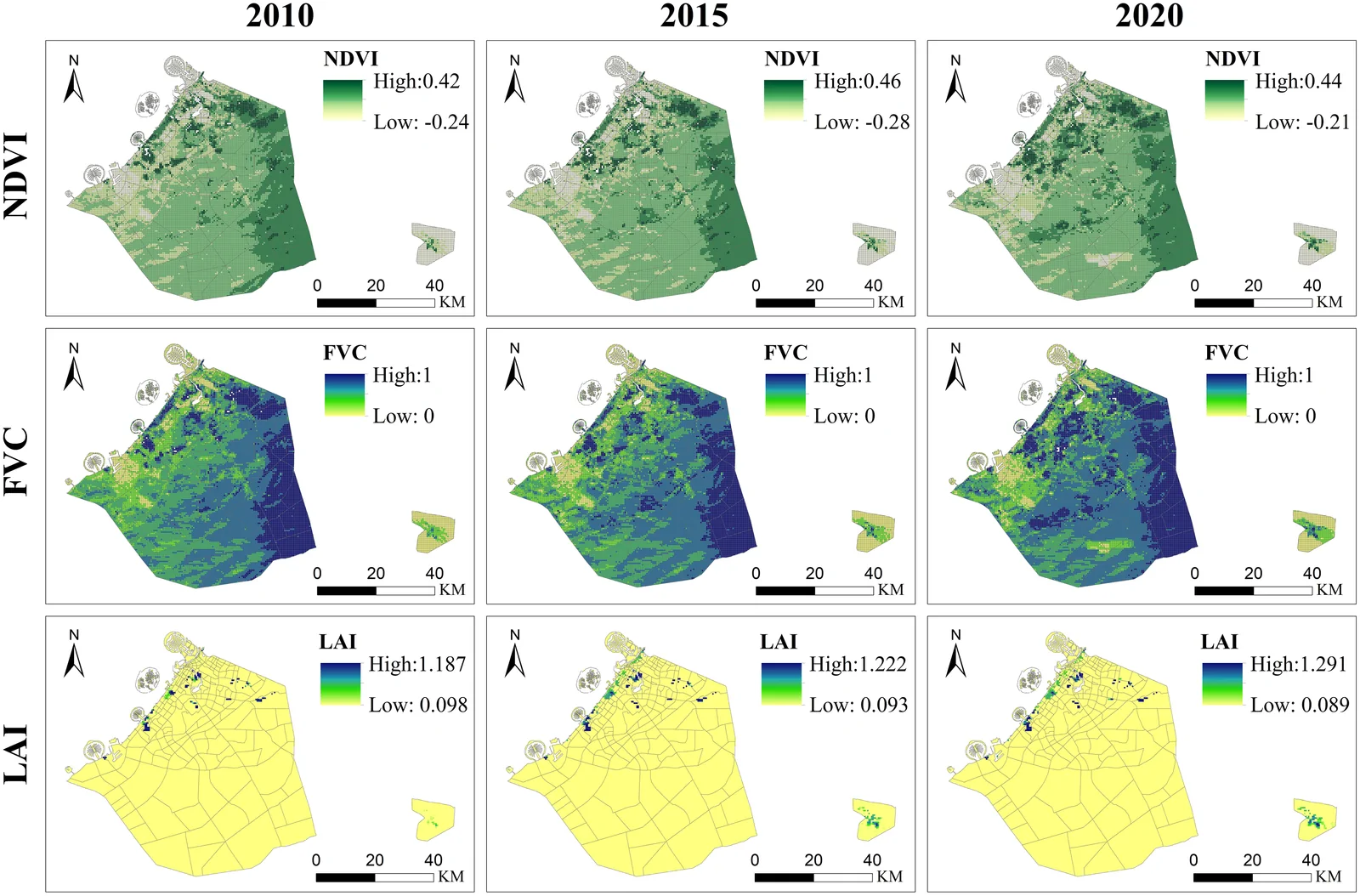
Spatial evolution of NDVI, FVC, and LAI (Boundary data: geoBoundaries, https://www.geoboundaries.org/, CC BY 4.0.).

LAI was substantially lower overall and exhibited a more concentrated spatial distribution. High LAI values were limited mainly to northern coastal greening nodes, several urban parks, and the Hatta exclave. The maximum LAI increased from 1.187 in 2010 to 1.291 in 2020. The contrast between the relatively broad distribution of NDVI and FVC and the limited distribution of high LAI values indicates that vegetation greenness or surface coverage did not necessarily correspond to a dense or structurally developed canopy. In desert cities, moderate vegetation-index values may reflect sparse or discontinuous vegetation, seasonal growth, or localised irrigated surfaces. LAI therefore provides additional information about vegetation structure that cannot be obtained from greenness or fractional cover alone.

The change maps in Fig 13 show that NDVI variation was highly localised. Areas with marked increases or decreases were mainly distributed in the northern coastal urban belt, development fringes, and several inland development nodes. Between 2010 and 2015, increasing and decreasing NDVI patches were interspersed. From 2015 to 2020, NDVI became more stable across much of the study area, although a notable decline occurred in the southern region. Changes in FVC covered a broader spatial extent. Declining patches were more widespread during the first period, whereas relatively extensive recovery occurred in the northern and central areas during the second period. Several inland areas, however, continued to experience declines. Changes in LAI were concentrated mainly in northern coastal greening nodes and the Hatta exclave, while most of the desert interior remained stable. These results indicate that vegetation change was spatially uneven and was closely associated with managed green-space development, vegetation renewal, and local environmental conditions.

**Fig 13:**
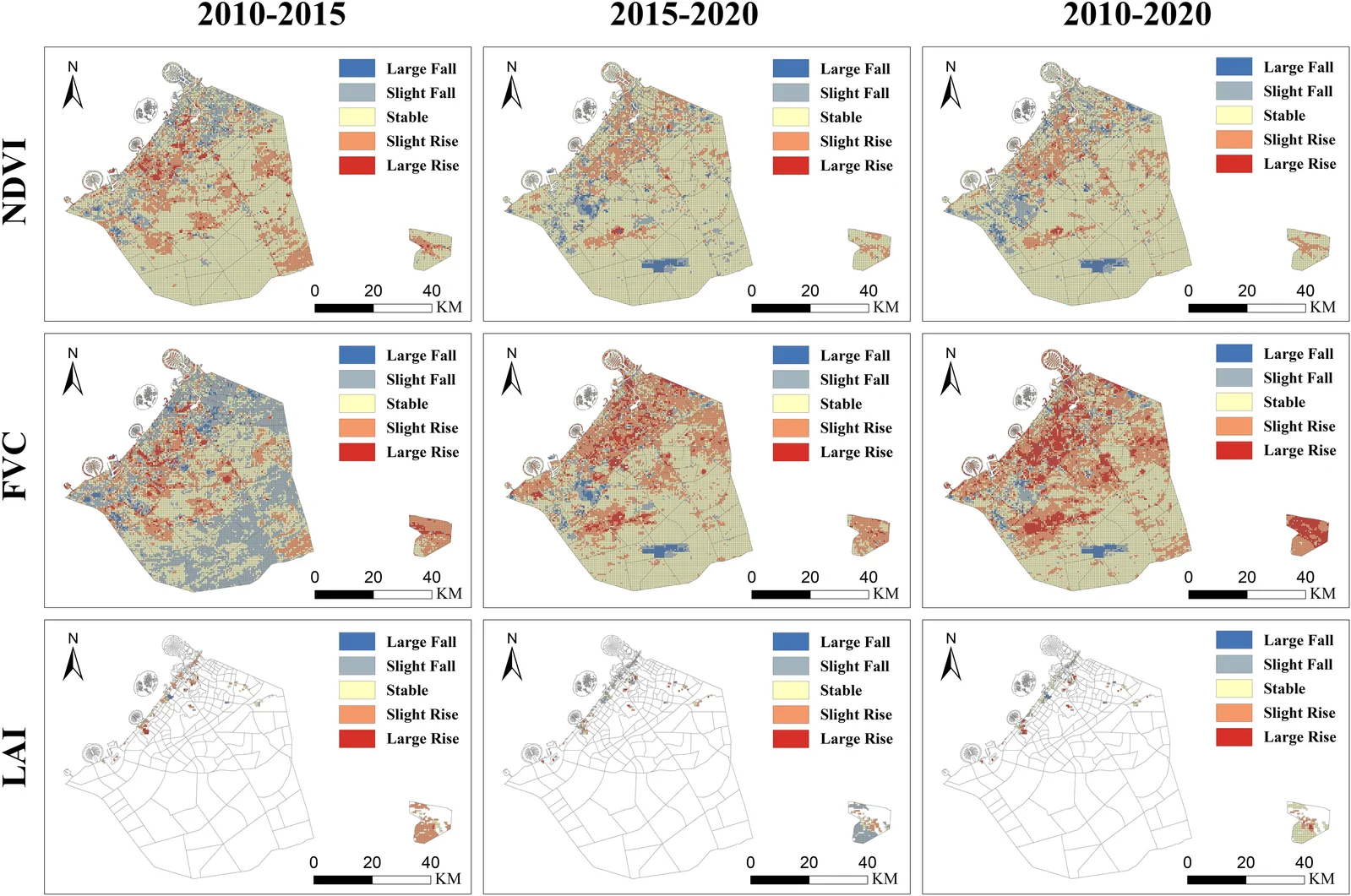
Spatial distribution of changes in NDVI, FVC, and LAI, 2010–2020 (Boundary data: geoBoundaries, https://www.geoboundaries.org/, CC BY 4.0.).

Table 2 quantifies the temporal differences among the three vegetation indicators. From 2010 to 2015, the mean annual dynamic degree of NDVI was 0.62%, while FVC and LAI declined by 0.15% and 1.35%, respectively. From 2015 to 2020, NDVI continued to increase at an annual rate of 0.51%, whereas the decline in FVC intensified to 0.79%. During the same period, LAI shifted to a positive annual rate of 1.36%. Across the full 2010–2020 period, the cumulative dynamic degrees of NDVI, FVC, and LAI were 1.15%, −0.93%, and −0.08%, respectively. The three indicators therefore described different aspects of vegetation change. NDVI increased consistently, suggesting an overall improvement in vegetation greenness. FVC declined over both intervals, indicating that the spatial extent or continuity of vegetation cover did not improve at the same rate. LAI decreased initially and subsequently recovered, resulting in a nearly stable cumulative change over the full decade. The results suggest that vegetation improvement in Dubai was expressed mainly through enhanced greenness and localised canopy recovery rather than through a citywide expansion of continuous vegetation cover. The reversal of LAI between the two periods may reflect vegetation maintenance, irrigation, or renewal in specific managed green spaces, but the spatial concentration of these changes indicates that the recovery was not representative of the entire urban region.

**Table 2:** Mean annual rates of change in NDVI, FVC, and LAI for individual periods.

Period	NDVI Dynamic Degree (%)	FVC Dynamic Degree (%)	LAI Dynamic Degree (%)
2010–2015	0.62	−0.15	−1.35
2015–2020	0.51	−0.79	1.36
2010–2020	1.15	−0.93	−0.08

Fig 14 confirms the different trajectories of the three indicators. NDVI increased during both five-year periods, although its rate of increase was slightly lower after 2015. FVC remained negative throughout the study period, with a stronger decline during 2015–2020. LAI showed the greatest fluctuation, declining by 1.35% during 2010–2015 and increasing by 1.36% during 2015–2020. The divergence between NDVI and FVC is particularly important. Although both indices were strongly correlated spatially, they responded differently over time, indicating that vegetation greenness and vegetation coverage should not be treated as interchangeable measures of urban greening performance.

**Fig 14:**
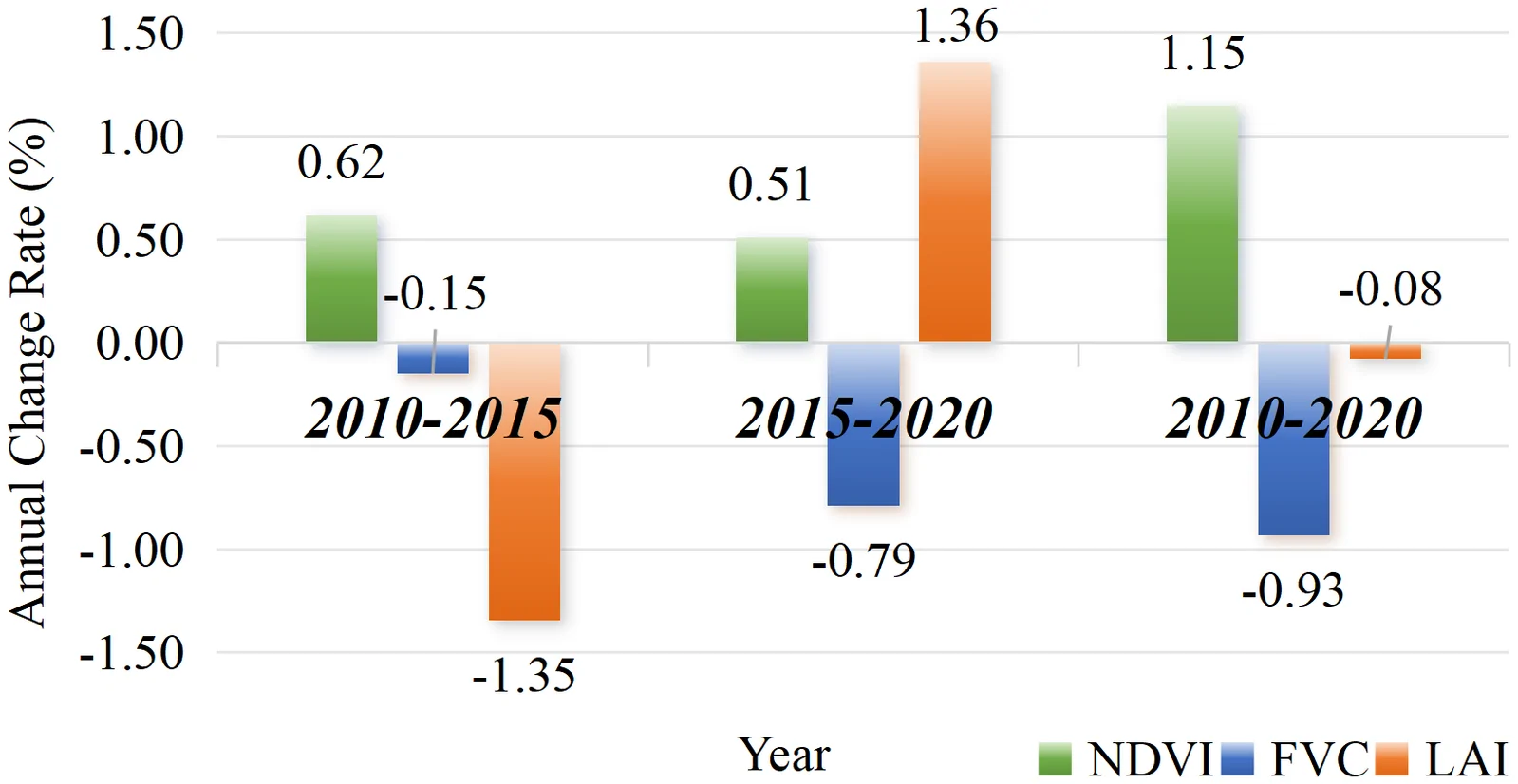
Grouped bar chart of the mean annual dynamic degrees of NDVI, FVC, and LAI, 2010–2020.

Fig 15 shows that Dubai retained a pronounced coastal–inland land-use gradient. Impervious surfaces formed a continuous built-up belt along the northern and northwestern Persian Gulf coast, surrounding the urban core, ports, artificial islands, and major transport corridors. Bare land dominated the central, southern, and southeastern areas and formed the principal desert matrix. Managed and sparse vegetation were embedded mainly within built-up areas, development fringes, and transport corridors, while water bodies and wetlands were concentrated along the coast and around artificial waterfront spaces. The broad spatial framework remained stable from 2010 to 2020, although built-up land gradually expanded along the northern coast and was accompanied by an increase in managed-vegetation patches. This indicates that urban expansion and artificial greening developed simultaneously within the same spatial corridor.

**Fig 15:**
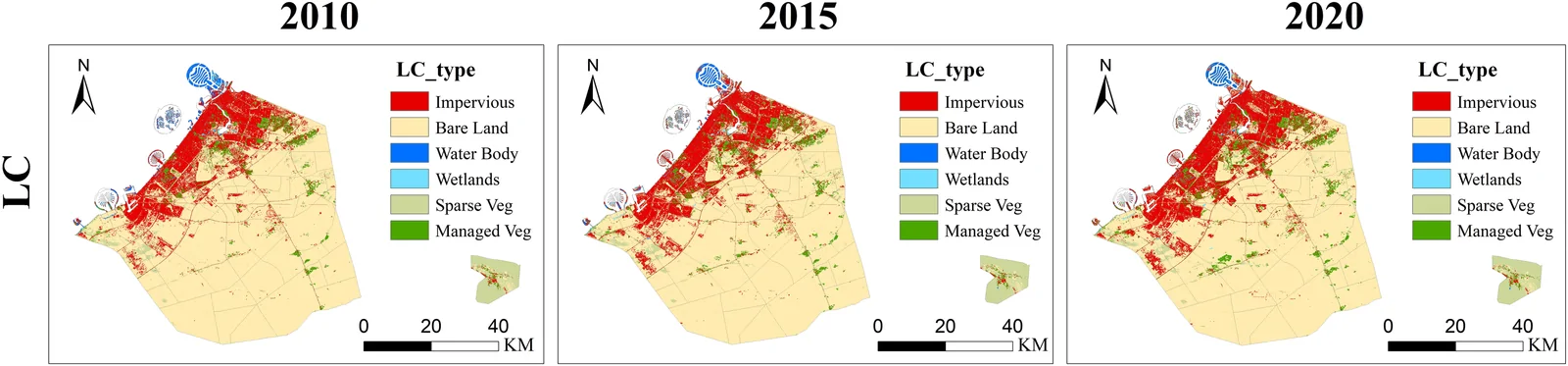
Land-use distribution in Dubai (Boundary data: geoBoundaries, https://www.geoboundaries.org/, CC BY 4.0.).

The land-use transition results in Fig 16 and Table 3 show that land-cover restructuring was most intensive from 2010 to 2015. During this period, 2,925.42 km² of managed vegetation remained unchanged, while 1,360.08 km² was classified as changing to water bodies, 575.54 km² to impervious surfaces, and 402.19 km² to wetlands. In addition, 591.57 km² of wetlands was classified as changing to water bodies. From 2015 to 2020, diagonal values became more dominant. A total of 2,875.61 km² of managed vegetation and 614.40 km² of impervious surfaces remained unchanged. The main off-diagonal transitions included managed vegetation to impervious surfaces, wetlands to water bodies, and managed vegetation to wetlands, with corresponding areas of 47.92, 43.33, and 37.25 km², respectively.

**Table 3:** Land-use transition matrix.

Year	Type	Impervious	Managed Veg	Sparse Veg	Bare Land	Water	Wetland
**2010-2015**	Impervious	256.34	281.2	2.47	0.07	0.6	0.95
	Managed Veg	209.17	2418.39	0.85	0.09	8.15	2.39
	Sparse Veg	19.39	17.24	23.04	3.38	0.75	0.04
	Bare Land	17.23	15.44	3.7	11.25	0.13	0.07
	Water	26.88	50.8	0.57	0.7	103.95	2.46
	Wetland	132.83	188.96	3.86	0.82	110.54	201.19
**2015-2020**	Impervious	614.4	24.74	0.64	0.92	7.76	13.38
	Managed Veg	47.92	2875.61	0.62	1.14	9.49	37.25
	Sparse Veg	3.41	1.15	26.75	0.99	0.97	1.22
	Bare Land	1.76	1.19	2.53	9.33	1	0.5
	Water	10.56	8.15	0.33	0.86	197	7.22
	Wetland	14.48	10.05	0.23	0.34	43.33	138.67
**2010-2020**	Impervious	182	0.82	0.76	36.11	15.83	541.63
	Managed Veg	4.03	2619.26	0.12	0.26	5.21	10.16
	Sparse Veg	7.34	19	25.75	3.4	7.25	1.1
	Bare Land	18.72	9.94	3.7	8.42	5.93	1.11
	Water	1.01	5.99	0.14	0.27	176.27	1.68
	Wetland	355.32	84.7	0.57	0.47	28.78	168.36

**Fig 16:**
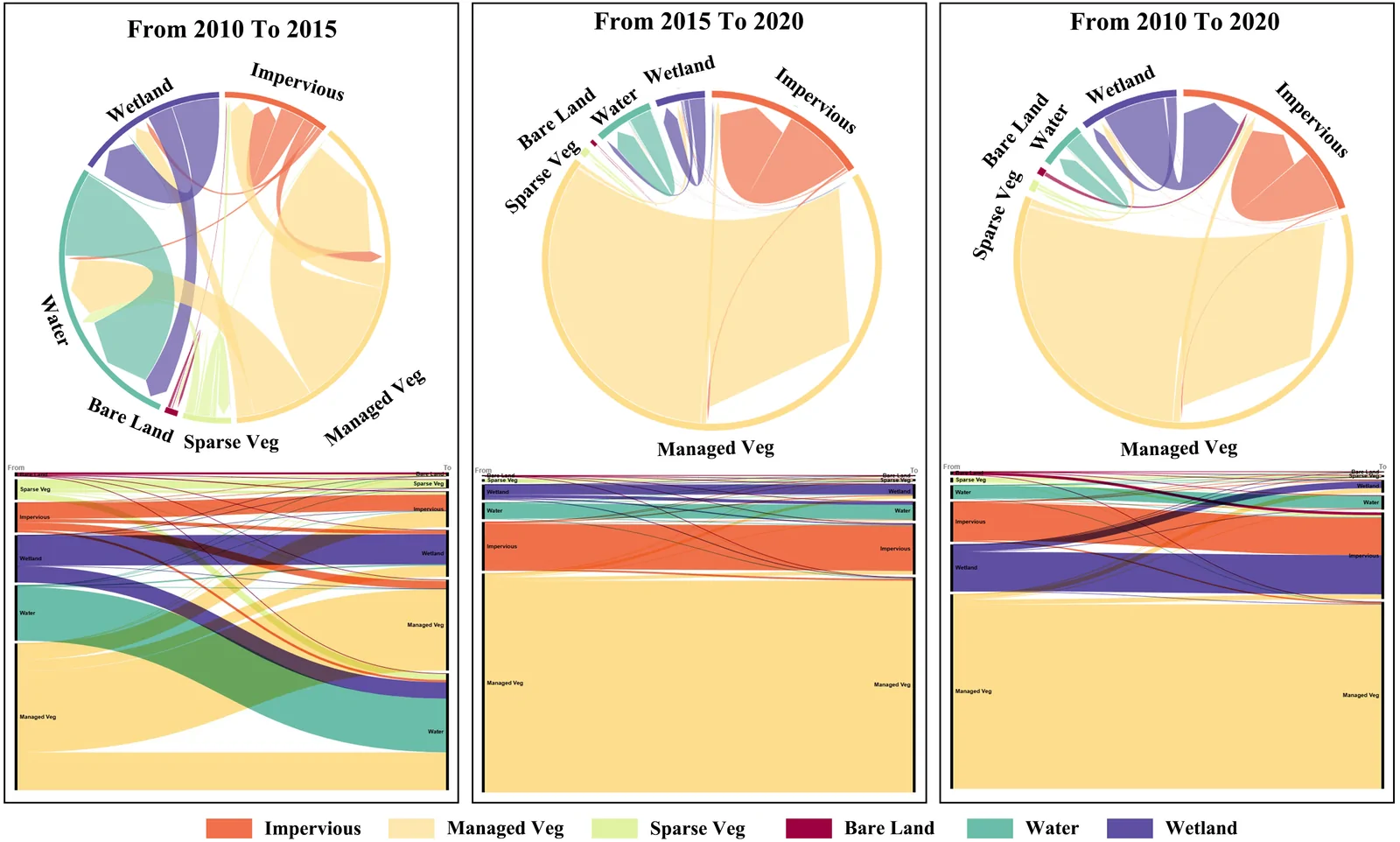
Land-use transition diagrams for Dubai, 2010–2015 and 2015–2020.

As shown in Table 3, the land-use transition matrix for Dubai during 2010–2015 reveals pronounced expansion of impervious surfaces and a substantial increase in managed vegetation, alongside notable mutual conversions between water bodies and wetlands, collectively indicating a highly active phase of urban land restructuring during this period. Specifically, the largest transitions into impervious surfaces came from managed vegetation (281.20), wetland (132.83), and water (26.88), while managed vegetation received considerable inputs from impervious surfaces (209.17), wetland (188.96), and water (50.80). Water and wetland also exchanged extensively, with 110.54 from wetland to water and 2.46 from water to wetland. In contrast, the transition magnitudes during 2015–2020 are markedly reduced, with all land-use categories exhibiting greater quantitative stability—particularly the sharply diminished influx into impervious and managed-vegetation classes. For example, the inflow to impervious surfaces from managed vegetation dropped to 24.74, and the inflow to managed vegetation from impervious surfaces fell to 47.92. The largest remaining transitions were internal retentions, such as 614.40 within impervious surfaces and 2,875.61 within managed vegetation, suggesting a deceleration of urban expansion and a progressive shift toward a relatively stabilised land-use configuration. Over the entire 2010–2020 period, the matrix further highlights a dominant transition from wetland to impervious surfaces (541.63) and from wetland to managed vegetation (84.70), along with a notable conversion from impervious surfaces to wetland (355.32), indicating complex long-term interactions among coastal, vegetated, and built-up land covers. Spatially, the changes remain concentrated in the northern coastal development belt, waterfront areas, and inland expansion fringes. Although the transition matrix alone cannot definitively distinguish actual land conversion from classification uncertainties, boundary shifts, or variations in image acquisition conditions, its temporal–spatial trajectories are highly consistent with the evolving trends of landscape-metric indices. This consistency further corroborates that the primary transformation processes during the study period involved coastal urban expansion, managed-vegetation development, and internal reorganisation of waterfront landscapes.

As shown in Table 4, from 2010 to 2020, the proportion of bare land decreased from 74.51% to 71.63%, and its edge density declined from 37.90 to 34.74 m/ha. However, patch density increased from 3.55 to 3.86 patches/100 ha, indicating that the reduction in total bare-land area was accompanied by increased fragmentation. The proportion of impervious surfaces increased from 15.27% to 16.99%, while edge density and patch density decreased by 0.86 m/ha and 0.43 patches/100 ha, respectively, reflecting the simultaneous expansion and spatial consolidation of built-up land. Managed vegetation exhibited the most pronounced changes, with edge density, patch density, and percentage of landscape increasing by 6.81 m/ha, 1.25 patches/100 ha, and 1.56 percentage points, respectively. This indicates that the expansion of urban greening was accompanied by substantial patch fragmentation. Sparse vegetation increased only slightly, whereas all three metrics for water bodies and wetlands generally declined, suggesting a contraction in the extent and boundary complexity of waterfront landscapes.

**Table 4:** Trends in ED, PD, and PLAND for different land-use types.

ClassName	Metric_Short	2010	2015	2020	Total_Change
Bare Land	ED (m/ha)	37.9	35.35	34.74	−3.16
Bare Land	PD (patches/100 ha)	3.55	3.74	3.86	0.31
Bare Land	PLAND (%)	74.51	72.89	71.63	−2.88
Impervious	ED (m/ha)	34.52	34.27	33.66	−0.86
Impervious	PD (patches/100 ha)	3.61	3.29	3.18	−0.43
Impervious	PLAND (%)	15.27	16.24	16.99	1.72
Managed Veg	ED (m/ha)	14.37	18.44	21.18	6.81
Managed Veg	PD (patches/100 ha)	2.85	3.74	4.1	1.25
Managed Veg	PLAND (%)	3.3	4.12	4.86	1.56
Sparse Veg	ED (m/ha)	14.26	15.65	15.58	1.31
Sparse Veg	PD (patches/100 ha)	3.45	3.85	3.91	0.45
Sparse Veg	PLAND (%)	5.24	5.49	5.4	0.16
Water	ED (m/ha)	2.38	1.39	1.03	−1.35
Water	PD (patches/100 ha)	0.55	0.54	0.5	−0.05
Water	PLAND (%)	1.31	0.86	0.77	−0.54
Wetland	ED (m/ha)	1.84	1.79	1.73	−0.12
Wetland	PD (patches/100 ha)	0.67	0.59	0.64	−0.03
Wetland	PLAND (%)	0.37	0.41	0.34	−0.03

As shown in Fig 17, the different land-use types exhibited marked differences across the three landscape metrics. Bare land and impervious surfaces consistently had higher edge density than the other land-use types, although both declined gradually over time. In contrast, the edge density of managed vegetation increased continuously, reaching 21.18 m/ha in 2020, indicating increasingly complex boundaries of artificially established green spaces. In terms of patch density, managed vegetation showed the largest increase, while bare land and sparse vegetation also increased to varying degrees; impervious surfaces, however, exhibited a gradual decline. Bare land remained the dominant land-use type in terms of percentage of landscape, although its share continued to decrease. Meanwhile, impervious surfaces and managed vegetation expanded steadily, indicating that land-use change in Dubai was primarily characterised by the gradual conversion of desert bare land into built-up areas and managed green spaces.

**Fig 17:**
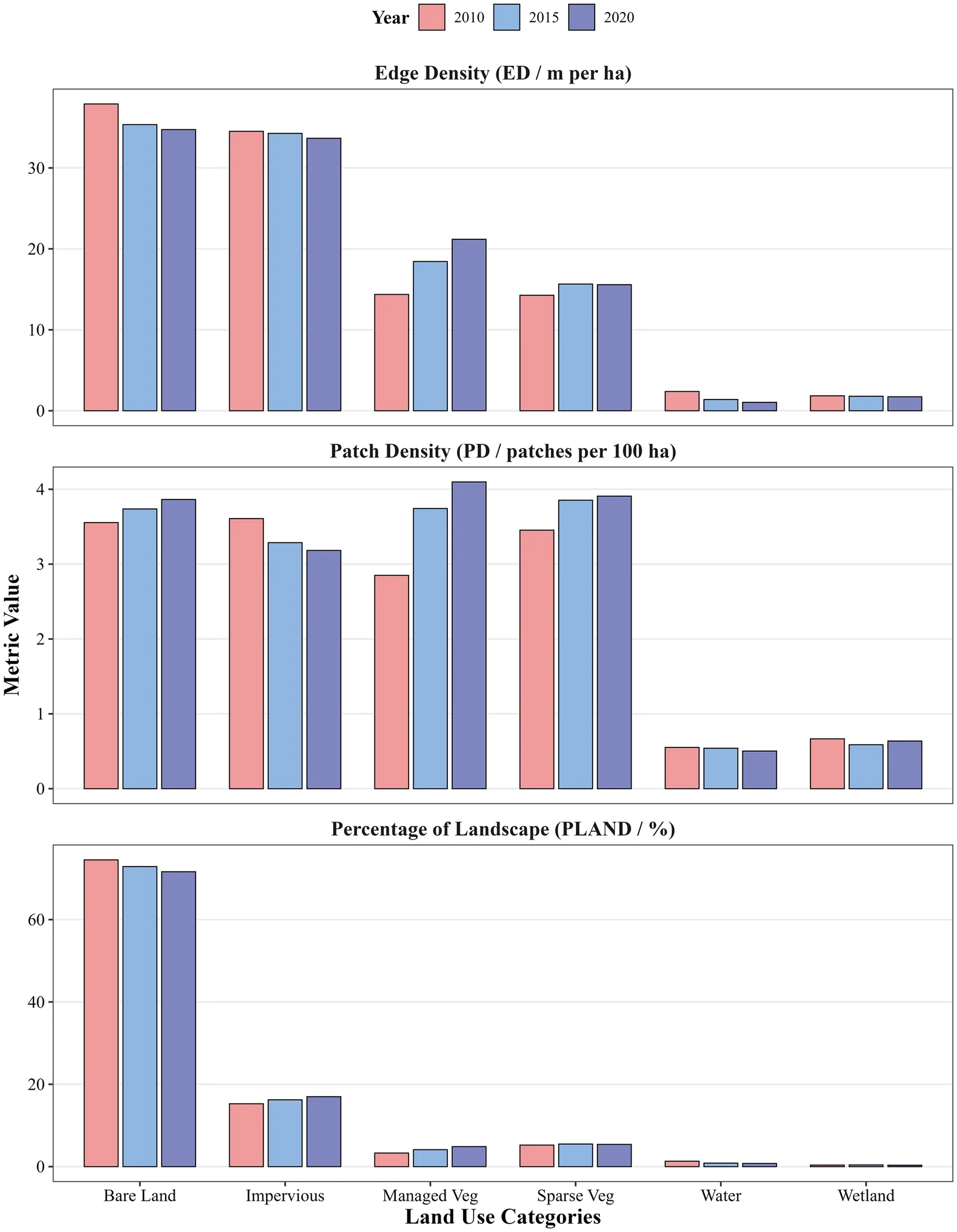
Temporal trends in ED, PD, and PLAND for different land-use types.

### 5.3. Correlation analysis

The correlation matrices in Fig 18 reveal strong relationships among several vegetation and landscape metrics. NDVI and FVC were strongly and positively correlated in all three years, with coefficients of 0.93, 0.89, and 0.89 in 2010, 2015, and 2020, respectively. This consistency indicates that the two indicators captured a common dimension of vegetation condition. However, their different temporal trajectories demonstrate that a strong spatial correlation does not mean that the two variables respond identically to land-use change. NDVI was more closely associated with greenness, whereas FVC represented the proportion and continuity of vegetated surface.

**Fig 18:**
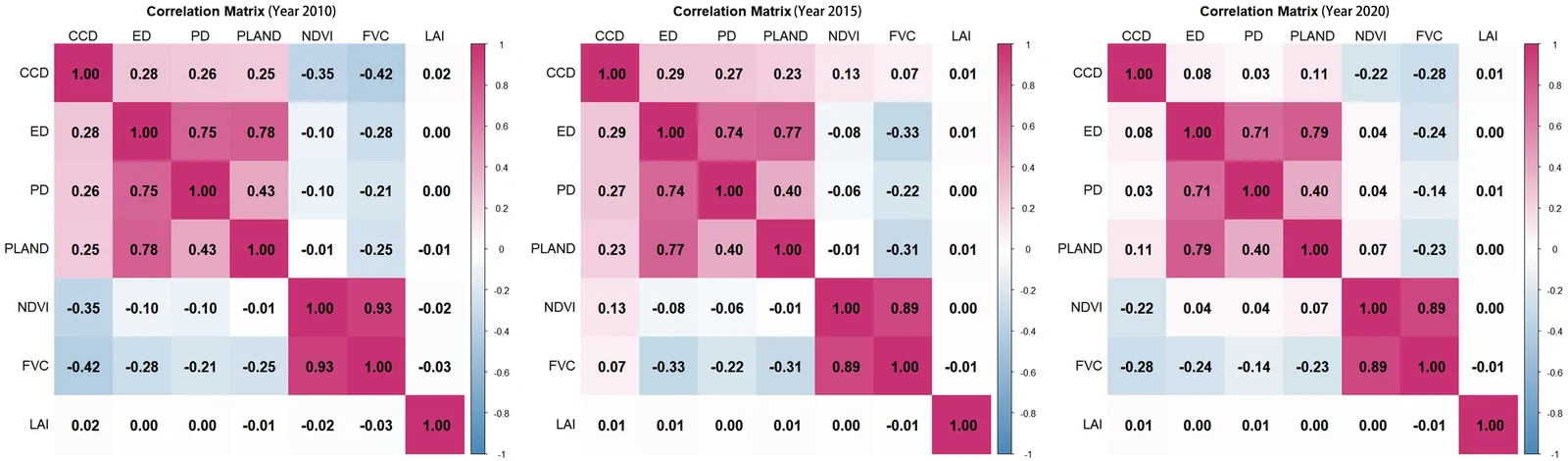
Correlation analysis of the study variables.

ED was also strongly related to PLAND, with correlation coefficients ranging from 0.77 to 0.79, and its correlation with PD consistently exceeded 0.70. These relationships indicate that landscape area, boundary complexity, and patch subdivision were closely connected. Larger or more widely distributed patches can produce greater total boundary length, while fragmented patches can simultaneously increase edge density and patch density. The high correlations among these metrics indicate that landscape structure should be interpreted as a set of interrelated properties rather than as a collection of completely independent indicators.

The relationship between the air–water coupling coordination degree and the explanatory variables varied among years. In 2010, coupling coordination was negatively correlated with FVC and NDVI, with coefficients of −0.42 and −0.35, respectively. It was weakly positively correlated with ED, PD, and PLAND. In 2015, the correlations between coupling coordination and most indicators weakened substantially. By 2020, the negative correlations with FVC and NDVI re-emerged. LAI remained weakly correlated with the coupling coordination degree and with the other indicators in all three years.

The negative correlations between vegetation indices and coupling coordination should not be interpreted as evidence that vegetation directly deteriorated atmospheric or water conditions. A more plausible interpretation is that vegetation was concentrated in developed or intensively managed areas where lower visibility, high irrigation demand, and spatially constrained water resources could coexist. Because the coordination degree integrates atmospheric clarity and soil moisture, the spatial presence of vegetation may be associated with a lower coordination value when its water requirement is not matched by the environmental conditions of the surrounding area. The weak association of LAI with the coordination degree further suggests that canopy leaf area alone did not adequately describe the spatial coupling between atmospheric conditions and water availability.

### 5.4. Relationships between landscape-pattern metrics and coupling coordination identified using the optimal-parameter geographical detector

The factor-detector results in Fig 19 indicate that the dominant explanatory factors changed substantially over time. In 2010, FVC had the highest explanatory power, with a q-value of 0.90, followed by NDVI with a q-value of 0.60. ED, PD, and PLAND had lower q-values of 0.42, 0.36, and 0.36, respectively. This result indicates that spatial differences in coupling coordination during the initial study period were primarily associated with vegetation-cover conditions. In 2015, ED became the dominant factor, with a q-value of 0.90. The explanatory power of PD and PLAND also increased to 0.73 and 0.60, respectively. The shift from FVC to ED suggests that landscape boundaries and patch structure became particularly important during the period of intensive land-use restructuring and urban expansion. Under these conditions, the spatial arrangement of green and non-green patches may have described environmental differences more effectively than vegetation amount alone. In 2020, FVC again became the strongest factor, with a q-value of 0.90, followed by NDVI at 0.59. The explanatory power of ED, PD, and PLAND declined substantially compared with 2015. LAI consistently had the lowest explanatory power, with a q-value of approximately 0.10 in all three years. The low and stable contribution of LAI indicates that leaf-area structure was spatially concentrated and therefore had limited capacity to explain the broad spatial differentiation of coupling coordination. Overall, the factor-detector results demonstrate that the dominant spatial correlate changed according to the stage of urban and landscape development. Vegetation coverage was more important in 2010 and 2020, whereas landscape-edge characteristics became particularly important in 2015.

**Fig 19:**
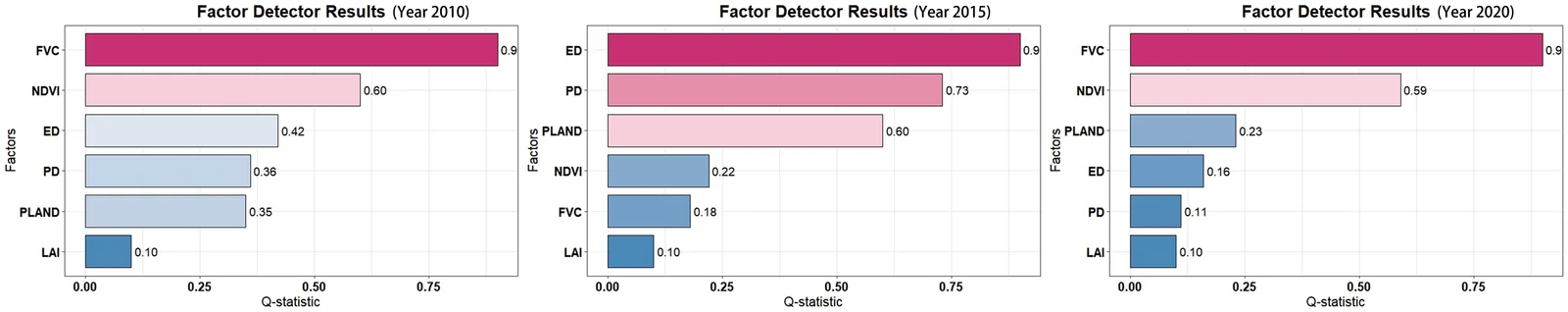
Results of factor detection.

Fig 20 further shows that the explanatory power of factor combinations was consistently greater than that of individual factors. In 2010, the strongest interactions were centred on FVC. The interaction between NDVI and FVC produced a q-value of 0.98, while LAI–FVC and PLAND–FVC produced values of 0.94 and 0.88, respectively. These results indicate that vegetation amount, greenness, canopy structure, and landscape proportion jointly described the spatial state of coupling coordination more effectively than any single indicator. In 2015, ED became the central interacting factor. The interaction between FVC and ED reached a q-value of 1.00, while NDVI–ED, PD–ED, and PLAND–ED reached 0.90, 0.85, and 0.80, respectively. This result indicates that the spatial influence of vegetation coverage was closely conditioned by the configuration of patch boundaries during the period of rapid landscape restructuring. In 2020, FVC again acted as the principal interaction factor. Its interactions with NDVI, PD, ED, and LAI produced q-values of 0.99, 0.98, 0.95, and 0.94, respectively. The interaction results demonstrate that coupling coordination was associated with the combined configuration of vegetation quantity and landscape structure. However, the q-value represents spatial explanatory power rather than the direction or magnitude of an effect. A high interaction value therefore indicates strong spatial stratification or joint explanatory capacity, but it does not demonstrate that the two factors exerted additive or causal effects. The repeated prominence of FVC–ED interactions suggests that the environmental meaning of vegetation coverage depended strongly on the complexity and fragmentation of its spatial boundary.

**Fig 20:**
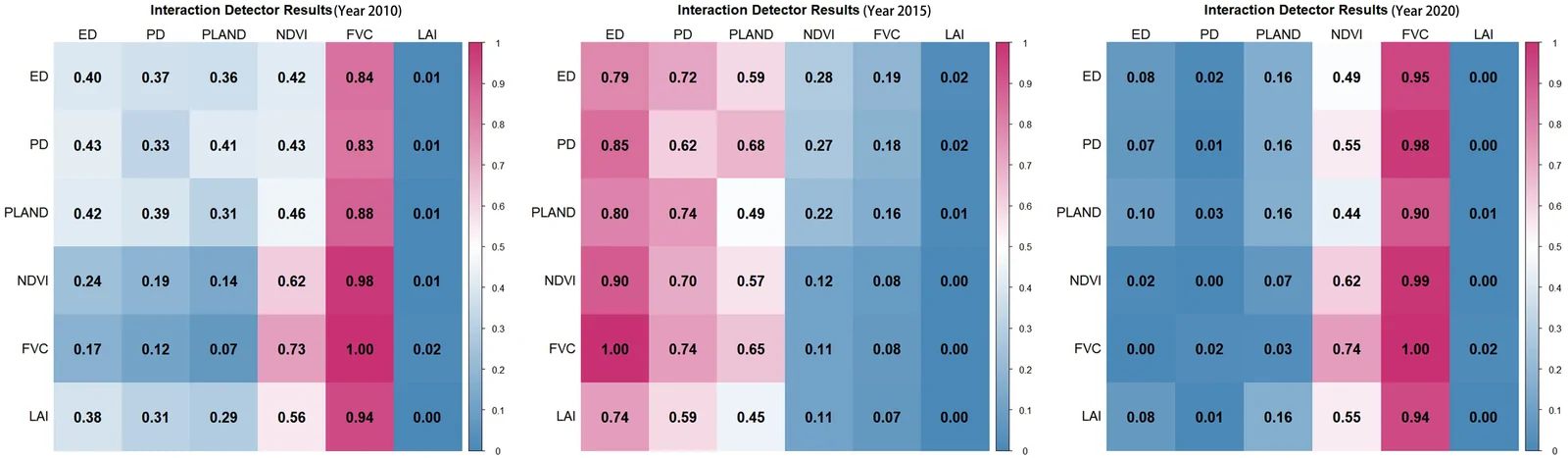
Results of interaction detection.

### 5.5. Relationships between landscape-pattern metrics and coupling coordination identified using spatial regression

#### 5.5.1. Ordinary least squares regression.

The OLS results in Table 5 indicate that FVC was significantly and negatively associated with the air–water coupling coordination degree in all three years. The coefficients were −0.3117 in 2010, −0.0236 in 2015, and −0.3589 in 2020, with all relationships significant at p < 0.001. The magnitude of the coefficient was substantially weaker in 2015, indicating that the global association between vegetation coverage and coupling coordination became less pronounced during the period when landscape structure and urban expansion were changing most rapidly.

**Table 5:** Coefficients and significance tests for variables in the global OLS regression models.

Variable	Statistic	2010	2015	2020
Constant	Regression coefficient	0.8832	0.5716	0.8232
	Standard error	0.0041	0.0045	0.0054
	(t)-value	215.160	126.394	153.056
	(p)-value	<0.001***	<0.001***	<0.001***
FVC	Regression coefficient	−0.3117	−0.0236	−0.3589
	Standard error	0.0052	0.0057	0.0072
	(t)-value	−60.327	−4.101	−50.074
	(p)-value	<0.001***	<0.001***	<0.001***
	VIF	1.367	1.377	1.424
ED	Regression coefficient	0.00021	0.00018	0.00007
	Standard error	0.00001	0.00001	0.00001
	(t)-value	39.366	33.377	11.457
	(p)-value	<0.001***	<0.001***	<0.001***
	VIF	2.185	2.141	2.312
PD	Regression coefficient	−0.00001	−0.00002	0.00007
	Standard error	0.00002	0.00002	0.00002
	(t)-value	−0.306	−0.860	4.576
	(p)-value	0.760	0.390	<0.001***
	VIF	2.001	1.929	2.074

ED had a significant positive association with coupling coordination in all three years. Its coefficient declined from 0.00021 in 2010 to 0.00018 in 2015 and 0.00007 in 2020. Although the positive direction remained stable, the declining coefficient indicates that the average association between boundary complexity and coupling coordination weakened over time. This change is consistent with the increasing complexity of managed vegetation and the changing spatial role of urban development boundaries. ED may therefore have captured different environmental configurations in different years, rather than representing a stable landscape function.

PD was negative and statistically insignificant in both 2010 and 2015, with coefficients of −0.00001 and −0.00002, respectively. In 2020, its coefficient became positive and significant, reaching 0.00007 with p < 0.001. This temporal shift suggests that patch number became more relevant after the expansion and subdivision of managed vegetation had become more pronounced. Nevertheless, the coefficient sign describes a statistical association with the composite coordination index and should not be interpreted as evidence that increasing patch number directly improved environmental conditions.

The variance inflation factors of all three retained predictors were below 2.5 in each year, indicating that severe multicollinearity was not present in the OLS models. This result is important because the correlation analysis showed strong relationships among several vegetation and landscape indicators. The retained variables therefore provided sufficiently distinct information for the estimated global associations, although the high correlations among the broader indicator set still require caution when interpreting individual coefficients.

The spatial distribution of standardised OLS residuals in Fig 21 reveals pronounced spatial clustering and clear interannual variation. In 2010, positive residuals were concentrated along the western and southwestern coast and in several northeastern areas, whereas extensive negative residuals occurred in the central-eastern and southeastern regions. In 2015, positive residuals dominated the northern coastal and central areas, while negative residuals were concentrated in the southern inland region and along several coastal margins. In 2020, a large negative-residual core emerged in the central urban area, while positive residuals were mainly distributed along the western coast, southern fringe, and several northern locations.

**Fig 21:**
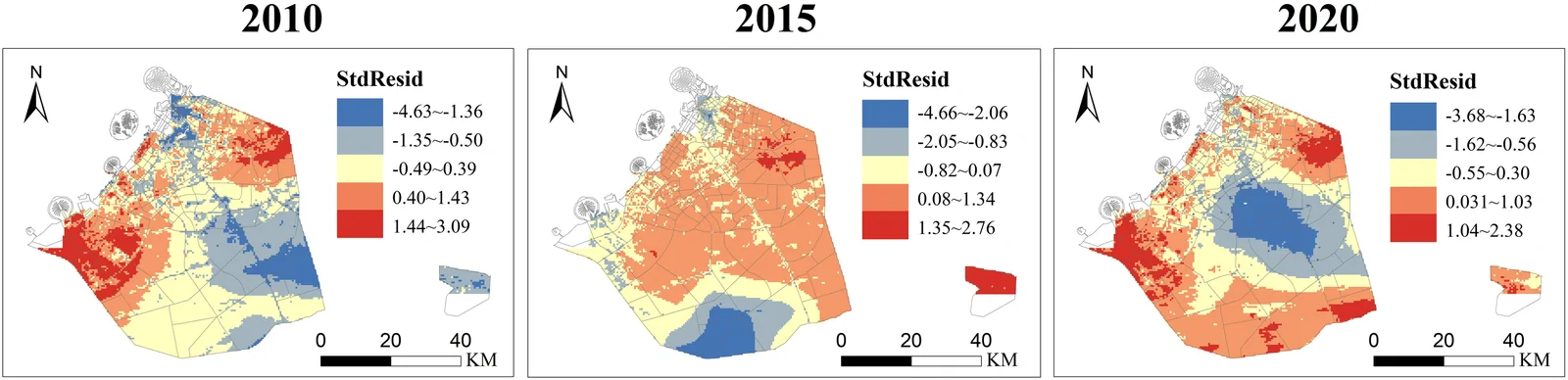
Spatial distribution of standardised OLS residuals (Boundary data: geoBoundaries, https://www.geoboundaries.org/, CC BY 4.0.).

These residual patterns indicate that a single set of global coefficients could not adequately represent the differences between intensively urbanised coastal areas, inland desert regions, and local development nodes. The residual clusters may reflect spatially structured conditions that were not fully captured by FVC, ED, and PD, including differences in land-use context, irrigation practices, microclimate, topography, atmospheric exposure, and urban morphology. The residual pattern therefore provides empirical support for applying a local spatial model. It does not, however, identify which unmeasured factor generated the remaining spatial dependence.

The OLS goodness-of-fit statistics in Table 6 further demonstrate the limitations of the global model. The sample size remained stable at approximately 15,700 observations in each year, with 15,695 observations in 2010, 15,696 in 2015, and 15,693 in 2020. The model achieved its highest explanatory power in 2010, with an R² of 0.3337 and an adjusted R² of 0.3336. The R² declined to 0.1020 in 2015 and partially recovered to 0.1681 in 2020. The adjusted R² values were nearly identical to the corresponding R² values, indicating that the difference was not caused by excessive model complexity or artificial inflation associated with the large sample size.

**Table 6:** Overall goodness-of-fit statistics for the OLS models.

Indicator	2010	2015	2020
Sample size (n)	15,695	15,696	15,693
Coefficient of determination (R^2^)	0.3337	0.1020	0.1681
Adjusted (R^2^)	0.3336	0.1018	0.1679
AICc	−32,091.46	−29,274.40	−20,575.28
Residual sum of squares (SSE)	118.87	142.26	247.52

The residual sum of squares was 118.87 in 2010, 142.26 in 2015, and 247.52 in 2020. The AICc values were −32,091.46, −29,274.40, and −20,575.28, respectively. The sharp reduction in R² in 2015 and the subsequent partial recovery in 2020 indicate that the global model performed unevenly across the three stages. Together with the clustered residuals, these results suggest that the relationship between landscape structure and air–water coordination was spatially nonstationary and changed over time.

#### 5.5.2. Geographically weighted regression.

As shown in Fig 22, local R^2^ values exhibited pronounced patch-based variation across Dubai. Areas of high and low explanatory power were interspersed, rather than forming a simple coastal–inland gradient. In 2010, relatively high-value patches were widely distributed along the southwestern coast and across the central and eastern inland regions, interspersed with numerous low-value corridors. In 2015, low-value areas expanded markedly, forming relatively continuous zones in the northern coastal built-up area and the central city, while high-value areas contracted mainly to the south and several peripheral locations. In 2020, high-value patches became more extensive again and displayed a multicentric distribution along the northern urban fringe, central area, and southwestern region, indicating a partial recovery in the local explanatory capacity of the landscape metrics. The Hatta exclave generally exhibited moderate to high explanatory power, although its temporal pattern did not fully correspond to that of the main Dubai territory.

**Fig 22:**
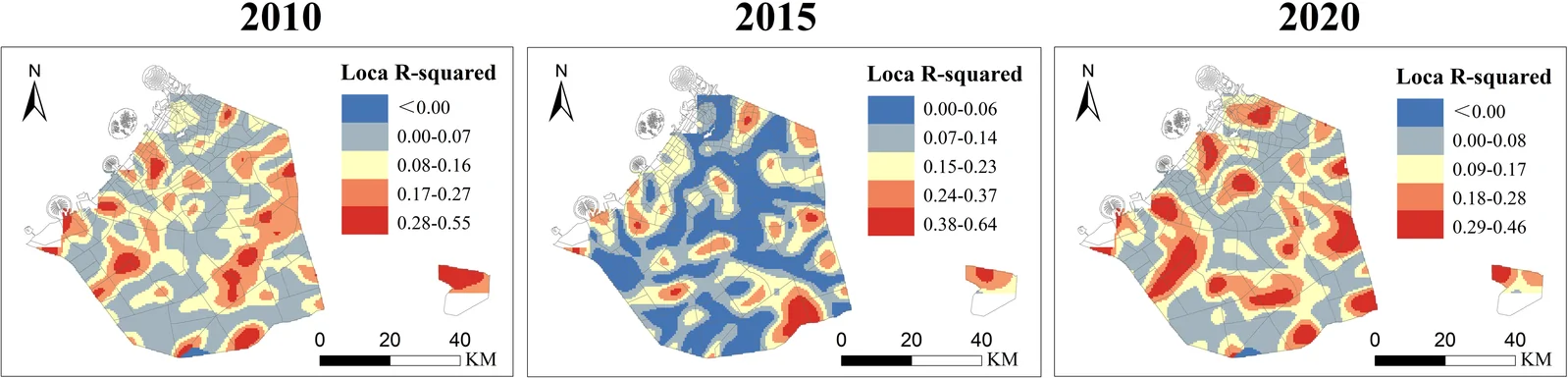
Spatial distribution of local R^2^, 2010–2020 (Boundary data: geoBoundaries, https://www.geoboundaries.org/, CC BY 4.0.).

As shown in Tables 7–10, the global R^2^ values of the GWR models reached 0.9906, 0.9874, and 0.9853 in 2010, 2015, and 2020, respectively, substantially exceeding those of the corresponding OLS models. The very high global R² values represented in-sample fitting performance. Spatial cross-validation produced substantially lower R² values, indicating that the model had moderate but limited out-of-sample predictive capacity.

**Table 7:** Global summary statistics of the GWR models, 2010–2020.

Indicator	2010	2015	2020
Effective sample size (n)	15,623	15,682	15,636
Global R^2^	0.9906	0.9874	0.9853
Adjusted R^2^	0.9905	0.9873	0.9852
Mean local R^2^	0.1141	0.1233	0.1373
AICc	−114,263.5	−109,847.2	−87,631.8
Cross-validation-selected number of neighbours	180	220	200
(p)-value of residual Moran’s (I)	0.127	0.153	0.086

**Table 8:** Descriptive statistics of local GWR coefficients in 2010.

Variable	Mean	Median	Minimum	Maximum	Standard Deviation	Positive Proportion	Negative Proportion
FVC	−0.0779	−0.0387	−1.6945	0.8720	0.1662	29.8%	70.2%
ED	0.000020	0.000009	−0.004047	0.004358	0.000097	59.1%	40.9%
PD	0.000011	−0.000007	−0.000986	0.001002	0.000245	45.1%	54.9%

**Table 9:** Descriptive statistics of local GWR coefficients in 2015.

Variable	Mean	Median	Minimum	Maximum	Standard Deviation	Positive Proportion	Negative Proportion
FVC	0.0461	0.0118	−0.8912	1.0812	0.2126	59.6%	40.4%
ED	0.000050	0.000008	−0.001691	0.008584	0.000272	64.2%	35.8%
PD	0.000001	−0.000010	−0.005413	0.002736	0.000493	44.0%	56.0%

**Table 10:** Descriptive statistics of local GWR coefficients in 2020.

Variable	Mean	Median	Minimum	Maximum	Standard Deviation	Positive Proportion	Negative Proportion
FVC	−0.0336	−0.0347	−1.5246	1.4080	0.2260	37.4%	62.6%
ED	−0.000028	−0.000011	−0.005397	0.000704	0.000147	41.2%	58.8%
PD	0.000026	0.000011	−0.002554	0.002472	0.000405	54.3%	45.7%

The local coefficients further revealed temporal shifts in the direction of the driving effects. In 2010, the mean FVC coefficient was −0.0779, and negative coefficients occurred across 70.2% of the study area. ED was predominantly positive, with positive coefficients covering 59.1% of the area. Although the mean PD coefficient was slightly positive, negative coefficients occupied 54.9% of the area.

In 2015, both FVC and ED became predominantly positive, with positive coefficients accounting for 59.6% and 64.2% of the study area, respectively, whereas PD remained predominantly negative. In 2020, FVC again exhibited a predominantly negative effect, with negative coefficients covering 62.6% of the area. ED shifted from positive dominance to negative dominance, with negative coefficients covering 58.8%, while PD became predominantly positive, with positive coefficients accounting for 54.3%. In addition, the standard deviation of the FVC coefficients increased from 0.1662 to 0.2260, indicating a continuous intensification of spatial variation in the effects of vegetation cover. Overall, the results demonstrate that none of the three indicators exhibited a stable or spatially uniform direction of influence across the entire study area.

The apparent global R² values of the GWR models were 0.9906, 0.9874, and 0.9853 for 2010, 2015, and 2020, respectively. However, these values were calculated from in-sample fitted values and should not be interpreted as measures of spatial predictive accuracy. The mean local R² values were only 0.1141, 0.1233, and 0.1373, indicating that the explanatory capacity varied considerably among locations. To further assess model generalisability, five-fold spatial block cross-validation was conducted. The cross-validated GWR R² values were 0.284, 0.216, and 0.318 for 2010, 2015, and 2020, respectively, with corresponding RMSE values of 0.071, 0.078, and 0.067 and MAE values of 0.053, 0.059, and 0.050. The corresponding OLS cross-validated R² values were 0.109, 0.081, and 0.137. Thus, GWR retained a higher out-of-sample explanatory capacity than OLS, although its predictive performance was substantially lower than its apparent in-sample fit. The cross-validation results also indicated that the original neighbourhood size of 50 observations was overly local for spatial prediction. In 2015, the spatial cross-validated R² increased from 0.108 with 50 neighbours to 0.216 with 220 neighbours and then declined slightly to 0.203 with 300 neighbours. The cross-validation-selected neighbourhood sizes were 180, 220, and 200 observations for 2010, 2015, and 2020, respectively. These results suggest that a moderate increase in neighbourhood size improved generalisability by reducing local coefficient instability while retaining sensitivity to spatial heterogeneity.

As shown in Fig 23, the local coefficients of FVC exhibited the most pronounced temporal shifts. In 2010, negative coefficients covered most of the northern coastal urban belt and the central inland area, while scattered positive patches occurred mainly in the south and southeast. In 2015, coefficients across the northern and central areas were predominantly close to zero, whereas relatively strong positive and negative values were concentrated along the southern desert fringe. By 2020, the negative effect had strengthened again, forming several concentrated patches in the central-eastern region, while positive coefficients were mainly distributed in the south and along parts of the urban periphery. This pattern indicates that the influence of vegetation cover on air–water coupling coordination differed substantially between coastal built-up areas and inland desert zones.

**Fig 23:**
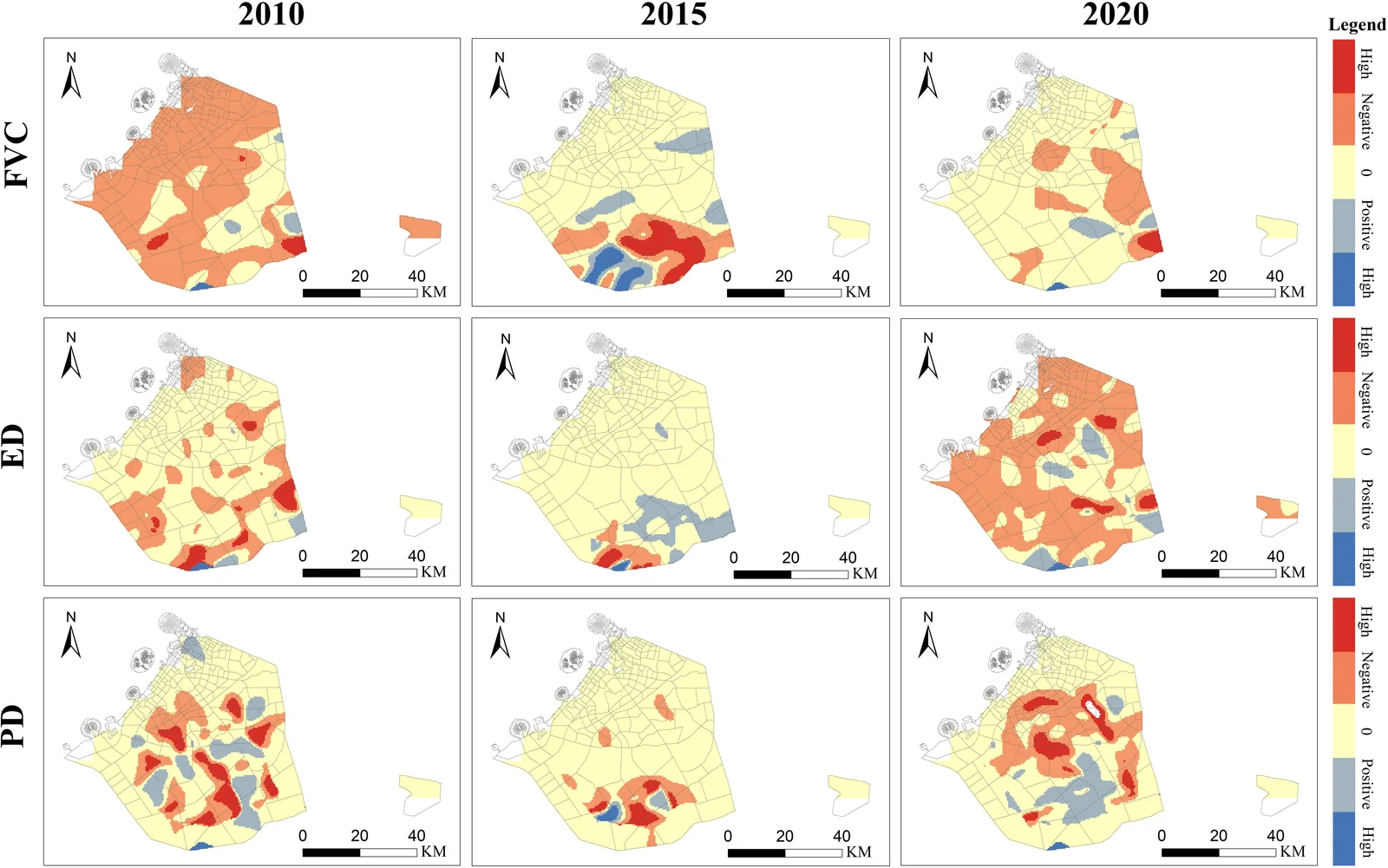
Spatial distributions of local regression coefficients for FVC, ED, and PD (Boundary data: geoBoundaries, https://www.geoboundaries.org/, CC BY 4.0.).

The ED coefficients were predominantly positive or close to zero in 2010 and 2015, while stronger positive and negative effects were concentrated mainly along the southern urban expansion fringe and in inland landscape transition zones. By 2020, the area characterised by negative coefficients had expanded considerably, covering much of the northern coastal periphery and central region, with only limited localised positive-value patches.

PD exhibited an interspersed pattern of positive and negative coefficients in 2010. Its areas of notable influence contracted to several southern locations in 2015. In 2020, positive coefficients expanded markedly across the central and southern regions, while annular or linear negative patches emerged along the northern urban periphery. Overall, the direction of influence of all three indicators varied by location and year, and no stable spatial response pattern was observed.

As shown in Fig 24, the continuous spatial clustering of the GWR standardised residuals was substantially weaker than that of the OLS residuals. Positive and negative residuals were distributed in a fine-grained, interspersed pattern across the northern coastal built-up area, the central urban periphery, and the southern desert interior. In 2010, the residual pattern was dominated by small positive patches, with scattered negative areas embedded among them. In 2015, several relatively strong negative residuals occurred along the southern fringe and in local coastal areas, but no continuous cluster extended across the study area. In 2020, negative-residual patches remained in parts of the central and northern urban areas, whereas positive residuals predominated in the peripheral regions.

**Fig 24:**
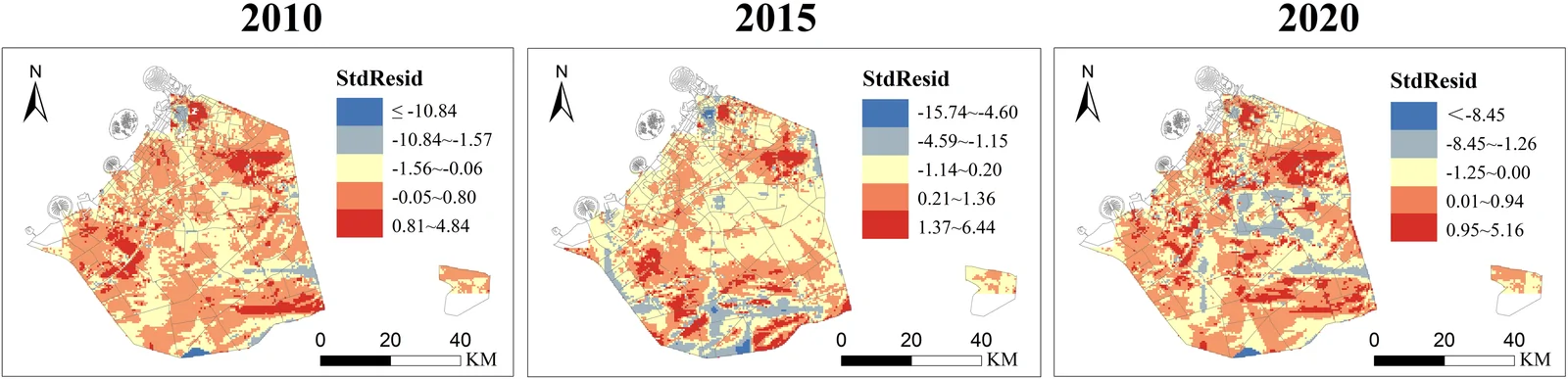
Spatial distribution of standardised GWR residuals (Boundary data: geoBoundaries, https://www.geoboundaries.org/, CC BY 4.0.).

Together with the residual Moran’s (I) test results for the three study years, these patterns indicate that residual spatial dependence was largely eliminated. This result also supports the use of GWR residuals as a diagnostic of model adequacy, because structured residual patterns may preserve information about unobserved social–ecological processes and spatial heterogeneity that are not captured by global predictors [26].

### 5.6. Small-sample field survey results

The small-sample field survey results were generally consistent with those derived from remote sensing and spatial modelling (Table 11). Coastal urban green spaces, central urban parks, and residential green spaces exhibited relatively high vegetation coverage and comparatively concentrated green patches, confirming the spatial concentration of managed vegetation in the northern coastal built-up area and major urban nodes. In contrast, green spaces in densely built-up areas and along central urban roads were predominantly distributed as linear strips or scattered patches along roads and block boundaries. This observation corresponds with the continuous increases in the edge density and patch density of managed vegetation.

**Table 11:** Small-sample field survey sites.

Site Type	Corresponding Photograph 1	Corresponding Photograph 2	Corresponding Photograph 3
Coastal urban green space	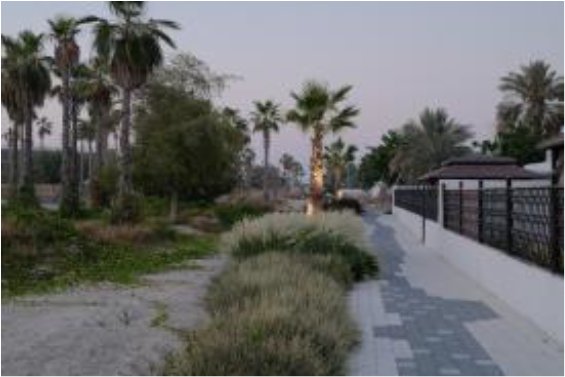	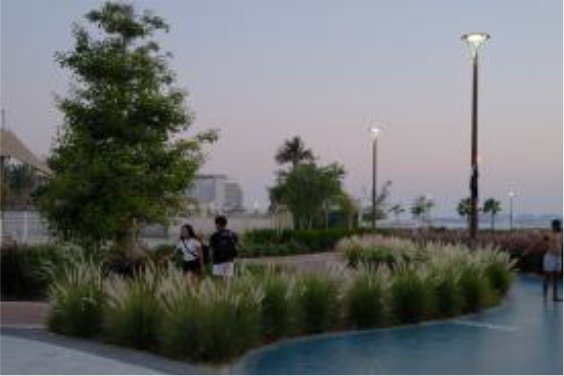	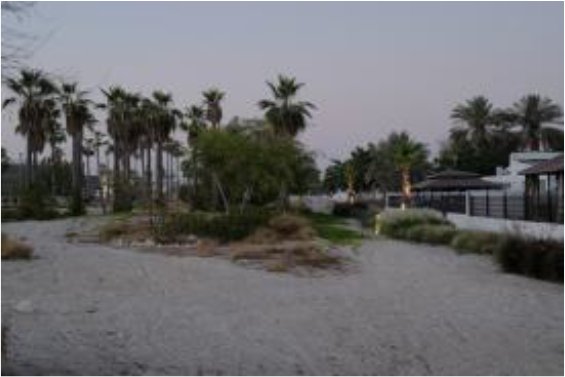
Green space in densely built-up areas	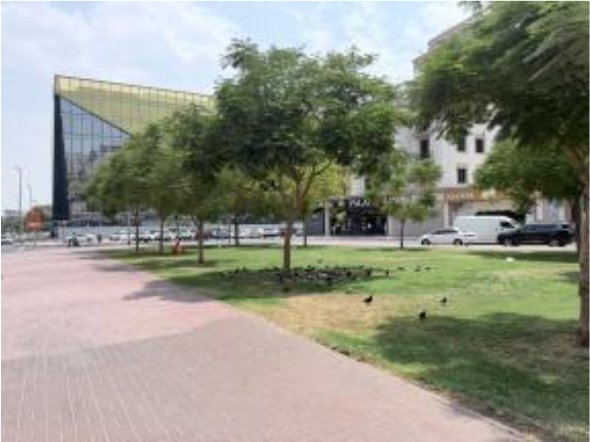	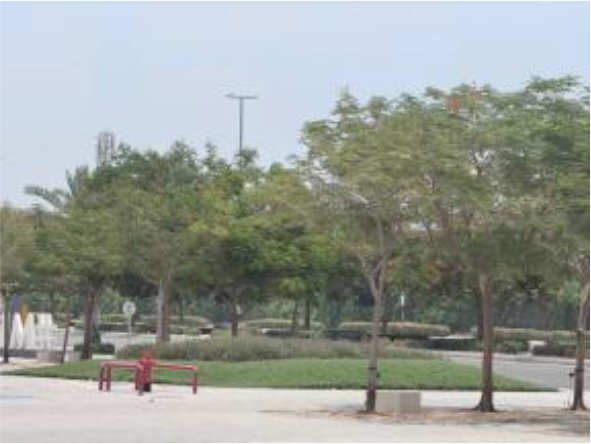	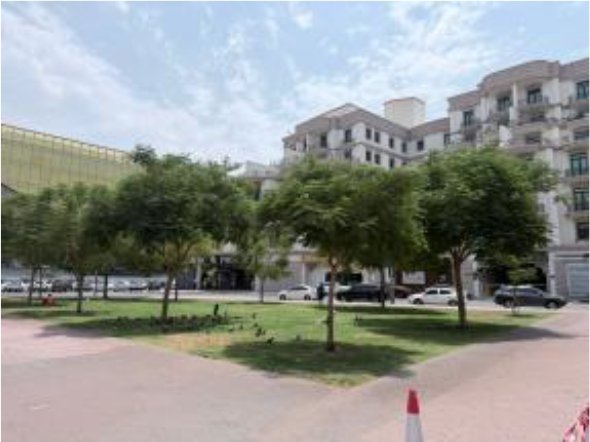
Large-scale managed greenbelt on the urban periphery	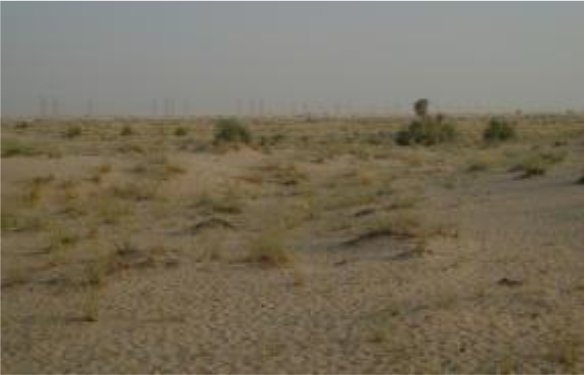	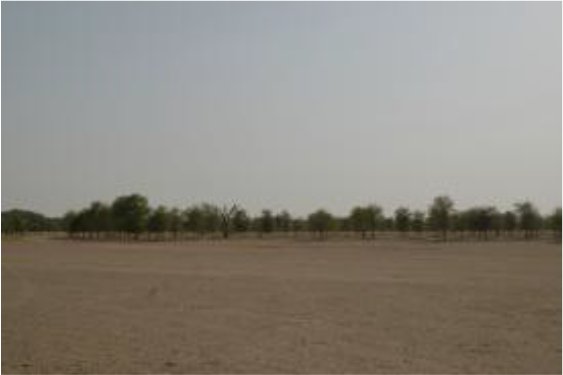	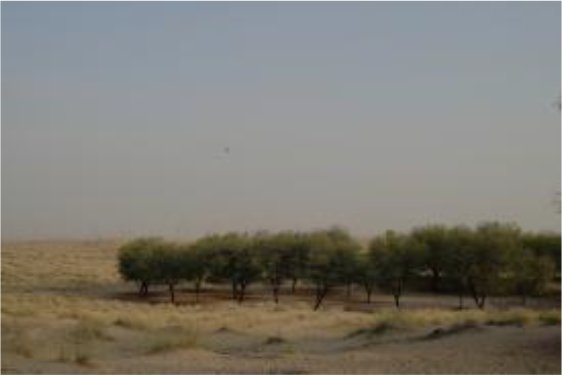
Roadside greening on the urban periphery	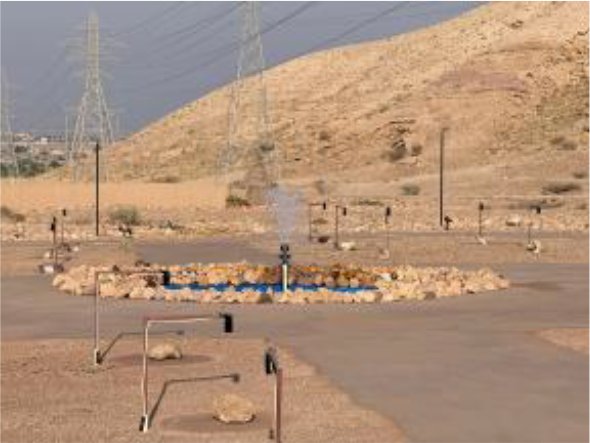	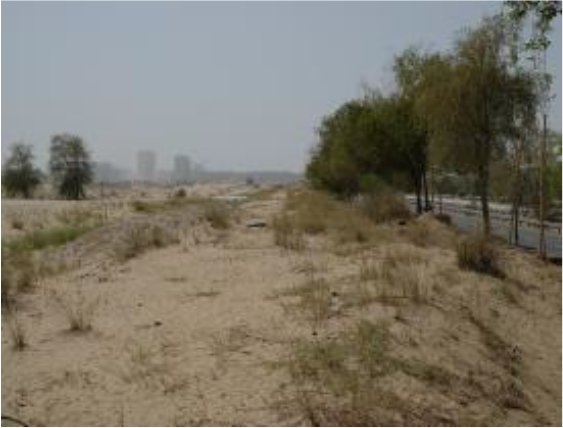	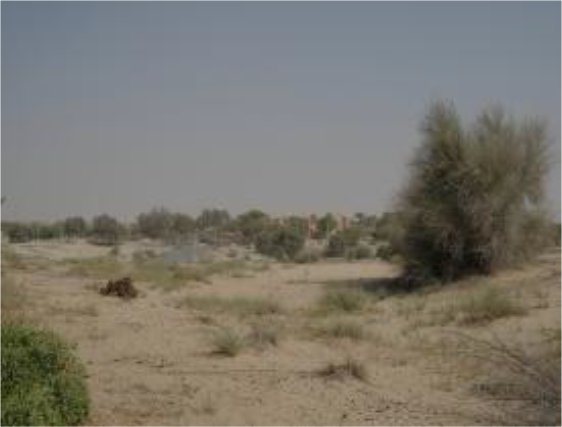
Natural landscape in remote suburban areas	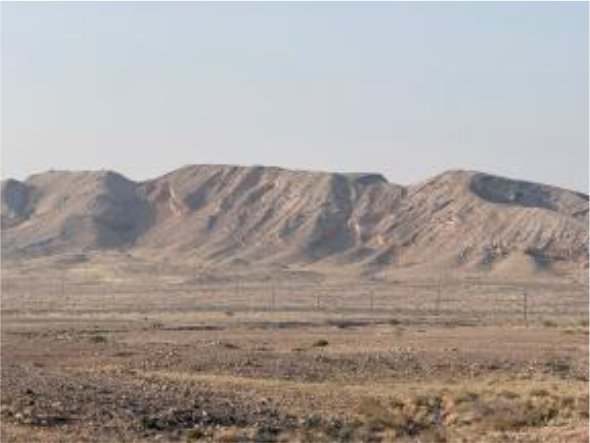	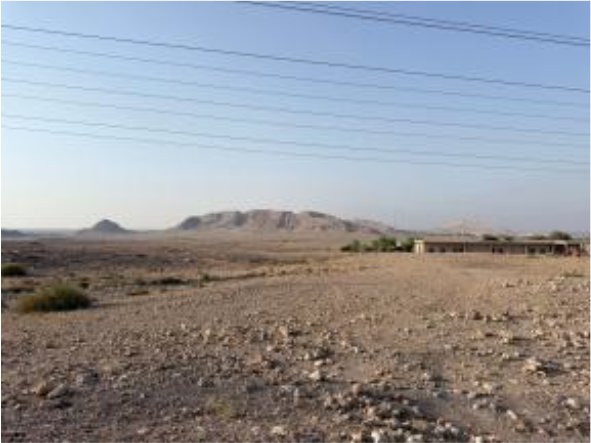	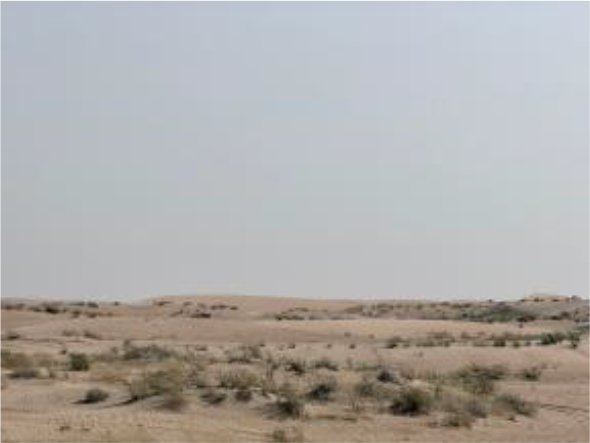
Roadside greening in the urban centre	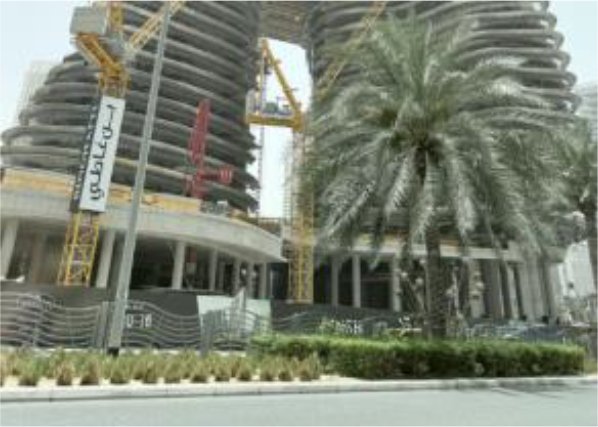	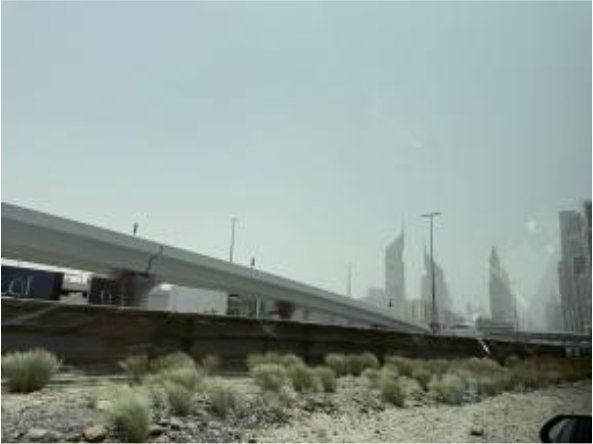	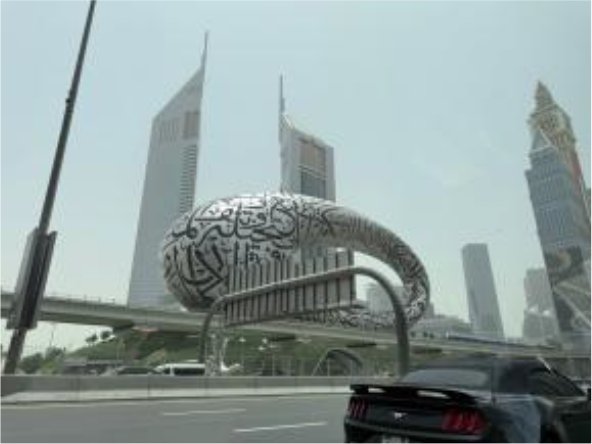
Central urban park	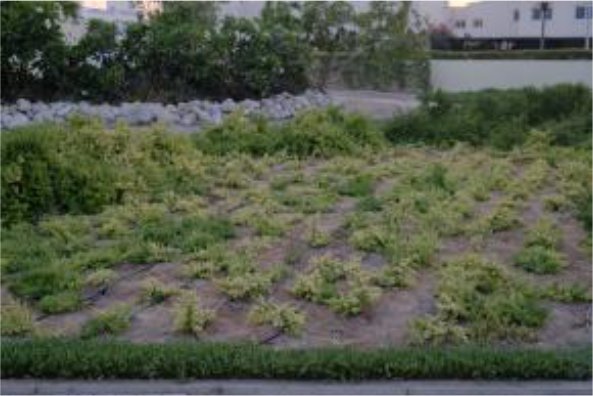	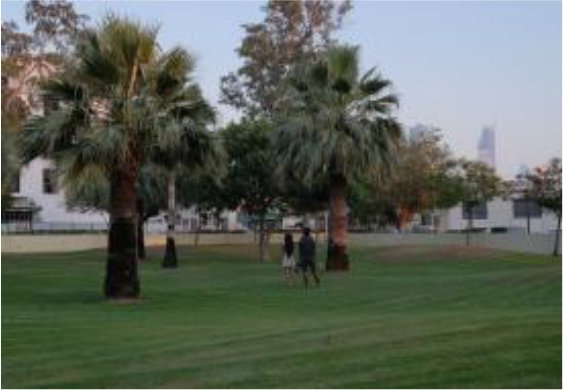	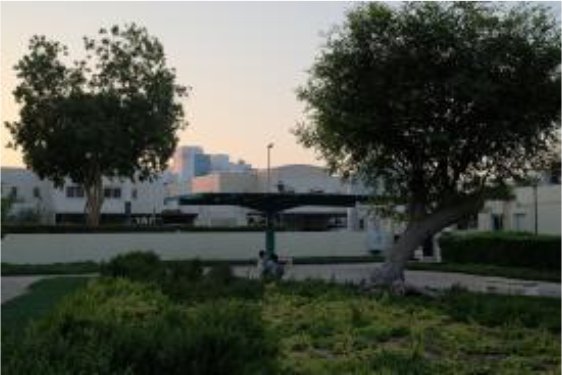
Residential green space	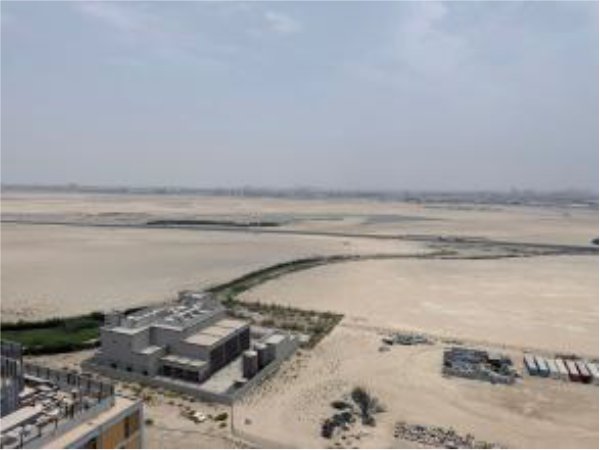	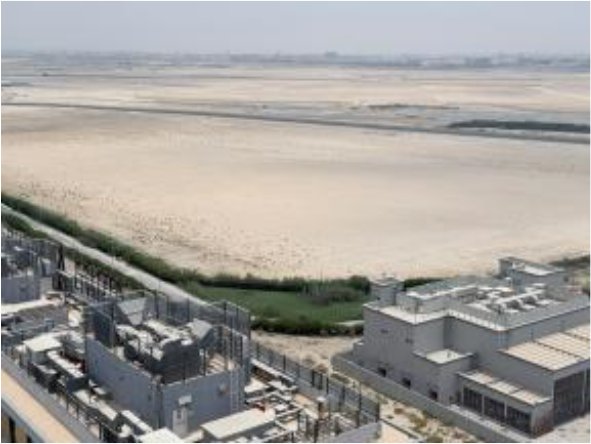	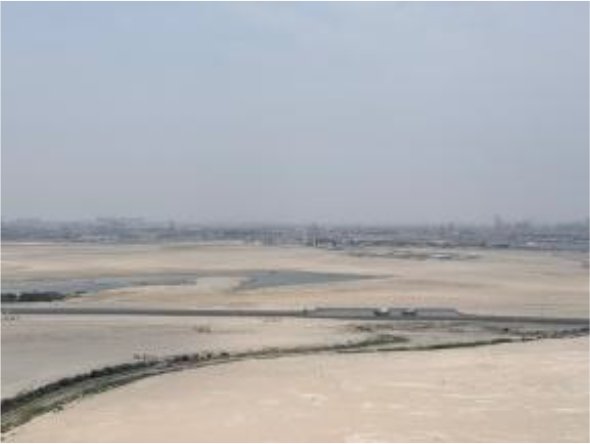

Large-scale managed greenbelts on the urban periphery exhibited relatively strong continuity and a substantial protective scale, whereas roadside green spaces in peripheral areas displayed a pronounced corridor pattern. These findings indicate that peripheral greening was primarily organised along urban expansion boundaries and major transport axes. By contrast, remote suburban areas remained dominated by bare land, sandy surfaces, and sparse natural vegetation, consistent with the extensive bare-land and low-vegetation-cover landscapes identified by remote sensing in central and southern Dubai.

The field observations therefore corroborated the large-scale patterns of coastal concentration, fragmentation in central urban areas, corridor-based distribution along the urban periphery, and bare-land dominance in remote suburban areas. The consistency in vegetation coverage, patch morphology, and greening configuration across the two analytical scales further supports the reliability of the study results. This correspondence further suggests that the ecological performance of urban greening depends on the functional connectivity and continuity of green-space networks, because fragmented patches may interrupt directional ecological flows and weaken network-level functionality, even when the total area of green space remains relatively large [27]. At a conceptual level, this cross-scale consistency also indicates that the air–water coordination state should be understood as an emergent property of interacting environmental subsystems, whose combined response cannot be adequately inferred from any single indicator in isolation [28].

## 6. Discussion

### 6.1. Factors shaping the current air–water resource coupling pattern

The results show that Dubai’s atmospheric and water-resource conditions did not evolve synchronously during the study period. Mean visibility generally declined from 2010 to 2020, whereas surface soil moisture decreased between 2010 and 2015 and subsequently recovered between 2015 and 2020. At the same time, areas with low air–water coupling coordination gradually shifted from the southeastern and southern parts of the study area towards the central urban area. This spatial and temporal pattern is important for the research question because it indicates that urban greening and water inputs did not produce a uniform improvement in the coupled environmental state.

The decline in visibility should be interpreted cautiously. Visibility is an indirect atmospheric optical indicator that may be affected by mineral dust, anthropogenic aerosols, relative humidity, fog, precipitation, wind conditions, boundary-layer structure, and atmospheric dispersion. Therefore, the observed decline cannot be attributed exclusively to dust storms or to changes in vegetation. In contrast, surface soil moisture is directly related to water availability but may also reflect irrigation, soil properties, rainfall distribution, and evaporation. The asynchronous changes in visibility and soil moisture therefore suggest that the atmospheric and water-resource subsystems were subject to different controlling processes.

The hyper-arid environmental background provides an important explanation for the observed spatial pattern. Dubai receives limited precipitation and experiences intense evaporative demand, particularly during the hot season. In inland desert areas, low soil moisture can reduce interparticle cohesion and make exposed surfaces more susceptible to wind erosion. Vegetation cover may stabilize the soil surface and reduce the exposure of fine particles, but the effectiveness of this process depends on vegetation continuity, plant structure, surface roughness, and local wind conditions. Recent satellite-based studies in the North African–West Asian dust belt have similarly emphasized the joint role of soil moisture and vegetation cover in constraining dust-emission potential, while also showing that their effects are nonlinear [29]. In the present study, these mechanisms are treated as plausible environmental explanations rather than as directly observed dust-emission processes because no event-level dust measurements were available.

The coastal–inland contrast further shaped the distribution of coupling coordination. High-density built-up areas, ports, airports, and transport infrastructure are concentrated along the northern Persian Gulf coast. The proportion of impervious surfaces increased from 15.27% in 2010 to 16.99% in 2020, whereas continuous bare land remained dominant in the central and southern inland areas. Although the total area of bare land declined, its patch density increased from 3.55 to 3.86 patches/100 ha. This suggests that roads, construction boundaries, and urban expansion increasingly subdivided exposed desert surfaces, thereby creating more interfaces between bare land, infrastructure, and developing urban areas. Such interfaces may increase local disturbance and road-dust resuspension even when the total bare-land area declines.

The spatial pattern of visibility was therefore likely shaped by a combination of regional atmospheric transport, local surface disturbance, construction activity, transport emissions, and ventilation conditions. Remote-sensing research in the Middle East has identified bare land as an important land-cover context for dust-emission sources, while transport infrastructure and human disturbance may further intensify local dust generation [30]. However, the present visibility data do not permit the relative contribution of these processes to be separated quantitatively.

The recovery of soil moisture after 2015 was also likely associated with artificial water inputs. Natural precipitation in Dubai is insufficient to sustain extensive urban vegetation over long periods, and managed green spaces in northern coastal built-up areas and along major transport corridors depend heavily on irrigation. The proportion of managed vegetation increased from 3.30% to 4.86%, while its edge density and patch density also increased. This indicates that newly established green spaces were frequently embedded within built-up areas as small and spatially dispersed irrigated patches. Irrigation can increase local soil moisture and maintain vegetation growth, but it does not necessarily improve visibility if transport emissions, regional dust transport, or restricted air exchange remain important.

The results therefore indicate a spatial mismatch between water inputs and atmospheric improvement [31]. Some areas with relatively high soil moisture still exhibited low visibility, whereas some inland areas with low soil moisture retained relatively high visibility during particular periods. This finding suggests that increasing irrigation or vegetation area alone may not be sufficient to improve the coupled environmental state. Effective planning should coordinate soil stabilization, vegetation structure, atmospheric ventilation, and water-resource allocation.

The divergence between atmospheric visibility, soil moisture, and vegetation indicators is consistent with previous studies showing that vegetation and soil moisture jointly affect dust-emission potential, while their effects vary with background climate, surface conditions, and human intervention. However, the present study extends this perspective by examining the spatial coordination between atmospheric clarity and near-surface water availability within an urban desert system. Unlike studies that focus on regional dust sources or individual irrigation interventions, our results show that coastal urban areas and inland desert areas may experience different environmental constraints despite belonging to the same administrative region.

### 6.2 Factors underlying the dynamic driving mechanisms

The land-use and vegetation results indicate that Dubai experienced simultaneous urban expansion and artificial greening, but these processes did not produce a simple improvement in vegetation conditions. From 2010 to 2020, the proportion of bare land decreased by 2.88 percentage points, whereas impervious surfaces and managed vegetation increased by 1.72 and 1.56 percentage points, respectively. This pattern indicates that part of the desert landscape was converted into built-up land and managed green spaces. However, the environmental meaning of this conversion depends on whether the newly established green spaces were continuous, structurally developed, and supported by an efficient water supply [32].

The vegetation indicators reveal different dimensions of greening change. NDVI increased over the study period, with a dynamic degree of 1.15%, whereas FVC decreased by 0.93% and LAI changed only slightly, with a dynamic degree of −0.08%. These results suggest that vegetation greenness improved locally, but the overall fraction of vegetated ground and the canopy structure did not improve correspondingly.

The increase in NDVI may reflect improved plant vitality in irrigated patches or the conversion of low-greenness surfaces into relatively greener patches. By contrast, the decline in FVC indicates that the spatial extent or continuity of vegetated ground did not expand at the same rate. The limited change in LAI further suggests that the development of vertical canopy structure remained constrained by water availability, planting form, climatic stress, or landscape fragmentation.

The distinction between NDVI and FVC is important because the two indicators represent related but different properties. NDVI primarily describes vegetation greenness and plant condition, whereas FVC describes the proportion of ground surface occupied by vegetation. The correlations between NDVI and FVC were 0.93, 0.89, and 0.89 in 2010, 2015, and 2020, respectively, indicating substantial information overlap. Nevertheless, NDVI is useful for identifying changes in vegetation condition, while FVC is more directly related to exposed-soil coverage and surface stabilization [16].

The high correlation also has implications for model specification. NDVI and FVC were retained in descriptive and GeoDetector analyses because their conceptual meanings are different. However, both variables were not entered simultaneously into the same multivariable OLS or GWR specification. This procedure reduces redundancy while preserving the ability to examine their separate spatial explanatory roles.

The landscape metrics further reveal that the expansion of managed vegetation was accompanied by fragmentation. The edge density of managed vegetation increased continuously and reached 21.18 m/ha in 2020. Patch density also showed the largest increase among the main land-use types. This indicates that new green spaces were primarily established as small patches, roadside strips, residential green areas, and development-fringe plantings.

Fragmented green spaces may produce local benefits, but they may also increase the length of vegetation–built-environment boundaries, intensify irrigation demand, and generate discontinuous ecological functions. Recent research on landscape connectivity emphasizes that the environmental performance of green infrastructure depends on patch arrangement, network continuity, and functional connections rather than total area alone. A fragmented green-space system may have a larger total edge length but a weaker capacity to stabilize exposed surfaces or guide airflow across larger spatial units.

The land-use transition results further suggest that the most active changes occurred during the 2010–2015 period, whereas land-use persistence increased from 2015 to 2020. This temporal difference provides a basis for interpreting the GeoDetector results. During the early period, changes in land-use composition and development boundaries were more pronounced. During the later period, the built-up and managed-vegetation structure became relatively more stable, and vegetation-cover conditions again became important in differentiating the coupling state.

The temporal shift from FVC dominance to ED dominance and then back to FVC dominance indicates that the importance of vegetation quantity and landscape structure changes with urban-development stage. This finding is consistent with recent spatial-planning studies emphasising that ecological performance depends on the interaction between landscape configuration and resource allocation. It differs from cross-sectional analyses that assume a stable hierarchy of explanatory variables across time. The comparison suggests that greening policies should be evaluated as stage-specific spatial interventions rather than as fixed citywide standards.

### 6.3. Sustainable landscape-design strategies

The GeoDetector results indicate that the dominant drivers of air–water coupling coordination changed with the stage of urban development. In 2010, FVC had the highest explanatory power, with a q-value of approximately 0.90, followed by NDVI with a q-value of 0.60. This pattern suggests that vegetation-cover conditions were closely associated with spatial differences in coupling coordination during the early study period, when extensive bare land remained around the urban periphery.

In 2015, ED became the dominant factor, with a q-value of approximately 0.90. The explanatory powers of PD and PLAND also increased to 0.73 and 0.60, respectively. This shift indicates that landscape structure became more important during the period of rapid urban expansion. The expansion of impervious surfaces and managed vegetation increased the number and length of boundaries between green spaces, built-up areas, and exposed surfaces. These boundaries may influence surface roughness, particle transport, airflow pathways, and the distribution of irrigation inputs.

In 2020, FVC again became the leading factor, with a q-value of approximately 0.90, while NDVI had a q-value of 0.59. The explanatory powers of ED, PD, and PLAND declined substantially. This may indicate that, after the built-up structure became relatively stable, differences in vegetation coverage again became more important than differences in landscape restructuring [33].

The interaction-detector results further indicate that the spatial differentiation of coupling coordination was jointly shaped by vegetation condition and landscape structure. The NDVI–FVC interaction produced q-values of 0.98 in 2010 and 0.99 in 2020, while the interactions between FVC and other landscape indicators also showed strong explanatory power. These results suggest that vegetation greenness, vegetation coverage, and landscape configuration should not be interpreted as completely independent processes.

For the 2015 FVC–ED interaction, the corrected q-value was strong joint spatial stratification of coupling coordination during the period of rapid urban restructuring [34]. It should not be interpreted as perfect prediction or causal determination. FVC describes the extent of vegetated surface cover, whereas ED describes the intensity of landscape boundaries. Their combined explanatory power may reflect the joint effects of exposed-soil stabilization, particle interception, surface roughness, airflow, and irrigation distribution.

The interaction results are consistent with previous research showing that the environmental performance of vegetation barriers depends on their height, thickness, porosity, leaf traits, continuity, and background wind direction [35]. Increasing vegetation quantity without considering its spatial structure may therefore produce weaker or opposite environmental effects in different urban contexts. The proposed planning strategies are also consistent with recent integrated conservation and restoration studies, which show that spatially explicit allocation can improve multiple ecosystem benefits under budget and resource constraints. The present results further indicate that this principle should be adapted to desert cities by jointly considering dust-source exposure, ventilation corridors, patch fragmentation, and irrigation requirements. The proposed strategies should be understood as a spatial-planning framework rather than as fixed engineering prescriptions. Their application requires local information on prevailing winds, irrigation systems, vegetation traits, soil conditions, and particulate-matter exposure.

### 6.4 Limitations and future research

This study has several limitations. First, mean visibility was used as an indicator of atmospheric clarity and has a different measurement meaning from direct particulate-pollution observations. Visibility is affected by aerosol loading, relative humidity, fog, haze, precipitation, wind conditions, and atmospheric mixing and dispersion. Consequently, variations in visibility cannot be interpreted directly as changes in PM10 or PM2.5 concentrations or as evidence of the occurrence and intensity of sand and dust storm events. This limitation is particularly relevant in Dubai, where humid coastal conditions, atmospheric aerosols, and episodic dust events may reduce visibility through different physical pathways. Future research should integrate ground-based PM10 and PM2.5 observations, aerosol optical depth, wind speed and direction, planetary boundary-layer height, relative humidity, and event-based dust indicators to distinguish changes in particulate loading from visibility reductions caused by meteorological conditions.

Second, the visibility–soil-moisture coordination index represents the joint variation of two environmental indicators and should not be interpreted as a complete atmospheric–water budget or a causal measure of greening performance. Additional variables, including irrigation volume, evapotranspiration, surface temperature, atmospheric aerosols, and groundwater conditions, are needed to represent the underlying processes more comprehensively.

Third, the spatial resolutions of the land-cover, vegetation, meteorological, and soil-moisture datasets differ. The analysis was therefore conducted using a common spatial support, and the coarser environmental variables were not treated as independent observations at the finer raster scale. Nevertheless, spatial aggregation may smooth local heterogeneity.

Fourth, GeoDetector, OLS, and GWR identify spatial associations and cannot by themselves establish causal relationships. Spatial cross-validation, bandwidth sensitivity analysis, and independent validation are therefore required to assess model generalisability.

Fifth, the field observations provide either quantitative validation only where matched measurements are available or qualitative contextual evidence where the survey consisted of photographs and visual observations. The field component should not be described as an independent quantitative validation if corresponding numerical observations are unavailable.

## 7. Conclusions

This study examined the spatial and temporal coordination between atmospheric visibility and near-surface soil moisture in Dubai, a water-constrained desert city. By integrating vegetation indices, land-use patterns, geographical detection, OLS regression, and GWR, the study evaluated how vegetation cover and landscape structure were associated with the coordination index across different development stages.

The results indicate that the coordination pattern was spatially differentiated and temporally dynamic. Atmospheric visibility generally declined during the study period, whereas soil moisture decreased initially and subsequently recovered. Low-coordination areas shifted across the study area, indicating that coastal built-up areas and inland desert areas were subject to different combinations of atmospheric and water-related constraints.

The analysis further indicates that vegetation cover and landscape structure did not have stable explanatory importance across time. FVC was the dominant individual factor in 2010 and 2020, whereas ED was dominant in 2015. The GWR results showed that the direction and magnitude of local associations varied across coastal built-up areas, urban expansion fringes, and inland desert areas. These findings support the central argument that citywide greening area is insufficient for evaluating environmental performance in desert cities.

The scientific value of this study lies in linking environmental coordination analysis with spatially differentiated landscape diagnosis. Its practical value lies in supporting separate strategies for exposed desert surfaces, development fringes, and densely built-up coastal areas. Peripheral areas should prioritise surface stabilisation and source control, development fringes should maintain continuous protective corridors, and densely built-up areas should balance vegetation-based interception with ventilation and irrigation efficiency.

These conclusions should be interpreted cautiously because visibility is not equivalent to particulate pollution or dust-storm intensity, the analysis is observational, the environmental datasets have different spatial supports, and the field validation is limited. Future research should integrate direct PM and aerosol observations, continuous dust-event records, irrigation data, wind-field simulation, and independent spatial validation.

The findings are most applicable to exploratory diagnosis and comparative spatial planning in water-constrained desert cities. They should not be used to estimate a universal causal effect of vegetation on dust or particulate pollution without direct monitoring and experimental verification.
